# Coarse-Graining of Slit-Confined Star Polymers in Solvents of Varying Quality

**DOI:** 10.1021/acs.macromol.5c01343

**Published:** 2025-10-31

**Authors:** Reyhaneh A. Farimani, Christos N. Likos

**Affiliations:** Faculty of Physics, 27258 University of Vienna, Boltzmanngasse 5, A-1090 Vienna, Austria

## Abstract

We investigate star polymers with varying functionalities and under varying solvent conditions confined within a slit geometry. Our approach involves accurately estimating and validating the effective interaction by directly computing the force between a pair of star polymers and comparing the radial distribution function from monomer-resolved molecular dynamics simulations with that obtained through Monte Carlo simulations using the effective interaction. Our findings reveal significant sensitivity in the radial distribution function to subtle variations in the tail of the interaction potential, particularly in dilute regimes. Furthermore, we employ a morphological model to analyze the interpenetration of the star polymers. We establish that solvent quality has minimal impact on the degree of interpenetration, whereas the star functionality affects it markedly, leading to enhanced faceting and reduced interpenetration for the number of arms grows. These results are particularly relevant for enhancing our understanding of polymeric materials’ rheological and mechanical properties.

## Graphical abstract

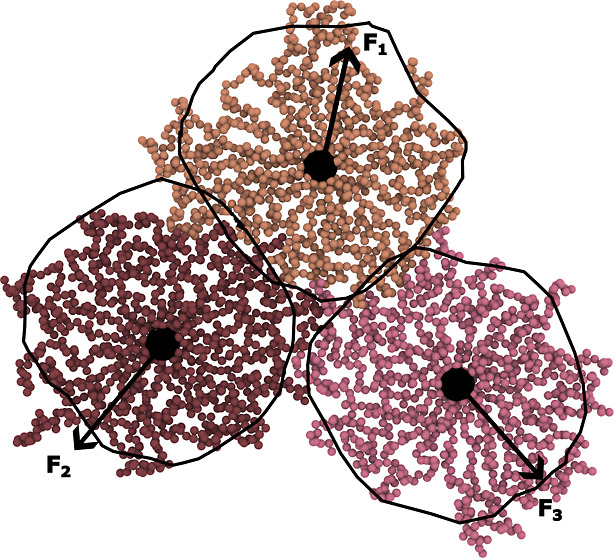

## 1 Introduction

Investigating colloids in confined environments has attracted considerable attention due to their scientific importance and practical applications. Colloids provide excellent models for studying two-dimensional materials, enabling researchers to verify theoretical frameworks that are challenging to observe in atomic systems. For instance, colloids have been pivotal in exploring the renowned KTHNY theory of phase transitions^1–3^ and in revealing intricate aspects of glassy dynamics within two-dimensional systems.^4,5^ Colloids under confinement offer significant industrial and technological applications, particularly in colloidal and polymeric films.

Star polymers represent a significant class of colloidal particles that have intrigued scientists for the past 50 years.^6^ The distinctive architecture of these ultrasoft particles provides a remarkable degree of tunability, establishing them as a natural link between polymers and colloidal particles and offering valuable theoretical insights into the behavior of soft matter.^7^ The three-dimensional phase diagram of these polymers reveals a diverse array of phases^8^ and showcases a variety of fascinating glassy states.^9–12^ Star polymers are easily synthesized and are increasingly utilized in various industrial and medical applications.^13–17^ Their structure features more reactive groups compared to a linear analog, making their nano- and microlayers particularly appealing for medical and biological uses.^18^ Additionally, their lightweight, stretchable, and impact-resistant characteristics position them as promising candidates for applications such as spacecraft coatings and bulletproof apparel.^19,20^ Recent research indicates that the unique star architecture may enhance the development of impact-resistant ultrathin polymer films, significantly improving energy dissipation mechanisms within these materials.^21,22^

The effective interaction between star polymers in athermal solvents has been studied in three-dimensional space, both theoretically and experimentally validated through numerical methods.^23,24^ This interaction is entropic, consistent with the polymeric characteristics of the system, and exhibits a logarithmic core. However, the shape of the tail poses challenges and depends on the polymers’ functionality.^25^ Paturej et al. have studied the star polymers under slit geometry using field-theoretical arguments and numerical computations.^26^ Chremos et al. studied the absorption of stars in two-dimensional layers.^27^ In two dimensions, the effective interaction between, once more, athermal star polymers has been explored using a field-theoretical approach, where Benhamou et al. conducted a theoretical analysis,^28^ and computationally by Egorov et al.,^29^ who confirmed these findings through molecular dynamics simulations for star polymers adsorbed on surfaces, having up to 12 arms. It has been found that for the case of full adsorption on surfaces, the effective potential is characterized by a logarithmic core and a Gaussian tail.^29^ While the short-range interactions of star polymers in two and three dimensions can be derived analytically, the specific form of the interaction tail remains unknown and is influenced by the system’s geometry and the polymers’ functionality. The precise tail of this interaction can significantly affect the system’s thermodynamics, as noted by Bos et al.^30^

The investigation of dense solutions of soft colloids has revealed that they undergo isotropic shrinking, followed by shape deformation and overlapping. There have been theoretical, numerical, and experimental studies of soft matter in three dimensions, utilizing different types of particles such as grafted nanocolloids and microgels as prototypes of soft colloids.^31–34^ Despite their differing perspectives, all these investigations treat each molecule as comprising a core and an interpenetrating soft shell. For instance, Midya et al.^33^ modeled the grafted nanoparticle as a core surrounded by extended, noninterpenetrating chains, along with a shell of unperturbed, interpenetrating chains. Their analytical model can predict the system’s behavior using a single parameter known as the overcrowding parameter. Moreover, the study of microgels typically considers these molecules as consisting of a core and a fuzzy soft shell.^35^ This deviation in polymeric systems enhances our understanding of the interplay between polymer deformation and interpenetration.

The present study extends previous considerations in various ways. In terms of geometry, we move away from a strict two-dimensional system to consider star polymers in slit confinement, exploring the ramifications this has on the tails of the interaction potential in particular. Our research focuses on rigorous confinement, utilizing a slit width comparable to monomer size to approximate a two-dimensional environment as closely as possible. Such strict confinement can be experimentally realized, as demonstrated in refs 36,37 . Moreover, we explore a broad spectrum of solvent qualities ranging from the athermal to the Θ-solvent. We examine the effects of solvent quality on the conformations and interactions of such confined star polymers. Finally, studying dense star polymer systems, we aim to uncover whether their interpenetration can be controlled through tunable parameters, providing insights into the design of advanced polymeric materials with tailored properties. Furthermore, these findings are crucial for verifying the validity of geometrical models, such as the liquid drop model, and for enhancing our understanding of the rheological and mechanical responses of soft matter systems.^38,39^

The details of the simulation and analysis of the computational work are presented in the Methods section that follows. Then we proceed with a systematic coarse-graining of the confined star polymers across different scales. In the first stage, we consider a single macromolecule, and we analyze, by scaling theory and simulation, its conformational properties for varying solvent quality, determining also the Θ-point of the polymer. Subsequently, we proceed with concentrated solutions, and we eliminate the monomers, reducing the stars in point particles interacting by means of effective pair potentials, which we model and validate against simulations for a broad range of concentrations, star functionalities, and solvent qualities. Finally, we also introduce a morphological coarse-graining of the solution, valid at all concentrations, which allows us to visualize the stars as deformable geometrical shapes, quantifying the degrees of interpenetration and faceting at arbitrary concentrations while at the same time maintaining in the picture information about the local monomer density in the solution.

## 2 Methods

We have studied a coarse-grained representation of star polymers consisting of *f* ∈ {10, 15, 20} linear arms, with each arm featuring *N* = 50 monomers. The arms are anchored to a central core, resulting in a total of *fN* + 1 monomers per star polymer. To prevent overcrowding near the anchoring region, and anticipating a quasi-2d confinement, the core size was scaled linearly with the functionality *f*, such that the system with *f* = 20 possesses the largest core with a radius *R*_core_ = 2σ. This modeling approach is consistent with experimentally synthesized star polymers, for instance, those prepared by functionalizing chlorosilane. In highly functionalized systems, the (dendritic) core size must be increased to alleviate steric constraints, and its diameter can reach several nanometers.^40^

In our simulations, we utilize an implicit solvent model, which accounts for solvent quality by incorporating an effective pairwise attraction between arms’ beads. In this model, we depict the nonbonded interactions between monomers of a macromolecule in a solvent, *V*_nb,λ_(*r*), using a Lennard-Jones potential with an adjustable depth^41^ controlled by the parameter λ as follows

1 $$V_{nb}\left(r,\lambda \right)=\{\begin{array}{l} 4\epsilon \left[{\left(\frac{\sigma }{r}\right)}^{12}-{\left(\frac{\sigma }{r}\right)}^{6}\right]+\epsilon \left(1-\lambda \right),,\text{if}\;r\leq 2^{1/6}\sigma ,\\ 4\epsilon \lambda \left[{\left(\frac{\sigma }{r}\right)}^{12}-{\left(\frac{\sigma }{r}\right)}^{6}\right],,\text{if}\;r>2^{1/6}\sigma . \end{array}$$

The impact of altering solvent quality is characterized by adjusting the parameter λ, which scales the strength of the attractive component of the pairwise potential. At λ = 0, a purely repulsive monomer–monomer potential arises (representing the athermal condition). An increase in λ signifies a decrease in solvent quality, akin to lower temperature, for most polymers. The nonbonded interactions between arms and core monomers, and core monomers with each other, are modeled using the Weeks–Chandler–Andersen (WCA) potential,^42^ expressed by eq 1 for the case of λ = 0. Bonding between sequential monomers along an arm, as well as between the first monomer in an arm and the star core, is modeled via the finitely extensible nonlinear elastic (FENE) potential^43^

$$V_{FENE}\left(r\right)=\frac{1}{2}kR_{\max}^{2}\;\ln\left[1-{\left(\frac{r}{R_{\max}}\right)}^{2}\right]$$ 2

In the eqs 1 and 2, *r* denotes the distance between monomers, ϵ and σ are chosen as the reference for the energy and length scales, respectively, with bead masses serving as the reference mass. The remaining parameters are fixed: *k* = 30ϵ/σ^2^, and *R_max_* = 1.5σ.

Star polymers are confined between two planar, parallel, repulsive walls, of size *L*_*x*_ × *L*_*y*_ placed parallel to the *x*–*y*-plane, and resulting thus in a slit geometry. The interaction between the walls and the monomers is modeled using the WCA potential, which operates perpendicularly to the walls in the *ẑ* direction. The slit width is set to *H* = 4σ, and periodic boundary conditions are applied in the *x̂* and *ŷ* directions, and the size in these directions was large enough to make sure no contact with the periodic image. A picture of a polymer between the two walls is shown in Figure 1. We have employed molecular dynamics (MD) simulations using the Large-scale Atomic/Molecular Massively Parallel Simulator (LAMMPS) package.^44^ The simulations were carried out in the *NVT* ensemble at a reduced temperature of *k*_B_ *T* = ϵ. Additional information is provided in the Supporting Information.

**Figure 1:**
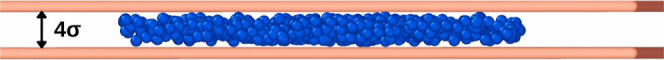
Snapshot of a star polymer in a slit.

To determine the effective interaction potential, we followed a procedure previously employed for this polymer architecture.^24,29,41^ Initially, we simulated two star polymers that were far apart, at a center-to-center distance *d* ≫ *R*_g_, where *R*_g_ is the individual star’s radius of gyration. After allowing the system to equilibrate, we gradually brought the well-equilibrated stars closer together, reducing the distance by small steps Δ*d* ≪ *R*_g_, waiting for the system to equilibrate again, and computing the average force on the core monomers. We repeated this process until the distance between the stars was comparable to 2*R_0_*, where *R_0_* is the radius of the core particle on which the *f* polymeric arms are grafted. This procedure was carried out for various values of λ and *f* to investigate the impact of solvent quality on the interaction force between the star polymers. To validate the effective interaction potential, we performed simulations of 32 star polymers at different concentrations ϱ, defined as

3 $$\varrho =\frac{32R_{g}^{2}}{L_{x}\times L_{y}}$$

We simulated at concentrations ϱ ∈ {0.05, 0.1, 0.15, 0.2, 0.25, 0.3}. The radial distribution function *g*(*r*) was calculated for the lowest concentration, ϱ = 0.05, where pairwise interactions dominate. This outcome was compared to Monte Carlo simulations of 1024-point particles interacting through the effective potential determined in the previous step. This comparison facilitated fine-tuning of the interaction’s tail, as high fluctuations had made direct MD results less reliable.

We conducted an investigation into the shape deformation and interpenetration of star polymer solutions. We utilized a surface mesh that encloses each star polymer to analyze these polymers in a continuous format. This process was carried out using the Alpha-shape method, available in the OVITO Python package.^45,46^

In this approach, the Delaunay tessellation is constructed from the set of monomer coordinates, decomposing the domain into tetrahedral elements. For each tetrahedron, the radius of its circumscribed sphere is computed; any tetrahedron whose circumsphere radius exceeds the probe threshold *r*_p_ = 8σ is designated as void and its triangular facets are discarded. Only those facets that separate a void tetrahedron from a solid tetrahedron (circumsphere radius ≤ *r*_p_) are retained, thereby automatically sealing all internal cavities smaller than the probe and yielding a fully closed, watertight surface mesh with Euler characteristic χ = 2.

The resulting surface mesh was then projected onto the *x*–*y* plane, creating a polygon. We calculated the overlap and area of this polygon using the SHAPELY Python package.^47^

## 3 Results and Discussion

### 3.1 Single Confined Stars in Solvents of Varying Quality

Although our star polymers are confined in a slit geometry, in what follows we treat them as two-dimensional since the slit width is much smaller than their overall size. We employ the Daoud–Cotton blob model for star polymers in two dimensions.^6^ This model was previously used for studying the star polymers in three dimensions and has shown to be successful.^48–50^ The model envisions every star as a succession of concentric blobs, each stretched on the plane along a wedge of opening angle 2π/*f*.^48–50^ Denoting with *s* the radial distance from the star center, a blob centered at *s* has radius ξ­(*s*) and contains *g*(*s*) monomers. The scaling relation between the two reads as

4 $$\xi \left(s\right)∼g^{\nu }\left(s\right)$$

with the scaling exponent

5 $$\nu =\{\begin{array}{l} 3/4,,good\;solvent,\\ 1/2,,\Theta ‐solvent. \end{array}$$

Let now ϕ­(*s*, *f*) be the local monomer density at *s*. The blobs are close-packed in the angular direction, thus there are *f*·*g*(*s*) monomers in a ring-shaped region of area π*s*ξ­(*s*) from the star center, so that we readily obtain

6 $$\phi \left(s,f\right)∼f\frac{g\left(s\right)}{s\xi \left(s\right)}$$

The close-packing condition along the radial direction, on the other hand, dictates that

7 $$\xi \left(s\right)∼\frac{s}{f}$$

Combining eqs 4, 6 and 7 above, we readily obtain

8 $$\phi \left(s,f\right)∼{\left(fs^{-1}\right)}^{2-1/\nu }$$

which, together with eq 5 yields

9 $$\phi \left(s,f\right)∼\{\begin{array}{l} f^{2/3}s^{-2/3},,good\;solvent,\\ f^{0}s^{0},,\Theta ‐solvent. \end{array}$$

The above relations also allow us to determine the scaling of the star radius *R* on *f* and the degree of polymerization *N* of each arm. Idealizing the star as a colloidal particle with a sharp boundary at *s* = *R*, the normalization condition on the monomer number reads as $2\pi \int_{0}^{R}s\phi \left(s,f\right)ds=Nf$ . Using eq 9 above and replacing *R* with the directly measurable radius of gyration *R*_g_ of the polymer, we readily obtain the scaling of the latter as

10 $$R_{g}\left(N,f\right)∼\{\begin{array}{l} f^{1/4}N^{3/4},,good\;solvent,\\ f^{1/2}N^{1/2},,\Theta ‐solvent. \end{array}$$

From eq 10, it becomes clear that not only each Daoud–Cotton blob but the whole star scales in size with *N* according to the corresponding exponents, ν = 3/4 or ν = 1/2, depending on solvent quality. The additional *f*-factors, *f* ^1/4^ for good solvent and *f* ^1/2^ for Θ solvent describe arm stretching due to crowding.

Equation 10 can be exploited to estimate the Θ-point of the model. Specifically, plotting the rescaled radius of gyration *R*_g_(*N*, *f*)/(*f* ^1/2^ *N* ^1/2^) against the interaction parameter λ for different *f* and *N* should reveal a common intersection, λ_Θ_. As shown in Figure 2, all functionalities converge near λ_Θ_ ≈ 0.78. It is important to stress that this value represents only an estimate of the Θ-point for finite-arm star polymers. In the asymptotic limit *N* → ∞, the Θ-point becomes topology-independent and can be rigorously defined via the vanishing of the second virial coefficient. For the present systems, variations in architecture (e.g., finite arm length and core size) may shift the apparent location of λ_Θ_. Our calculation is therefore intended primarily as an operational comparison to bulk star-polymer results rather than a universal determination. For reference, in three dimensions the same model yields λ_Θ_ = 0.48,^41^ consistent with the expectation that confinement enhances effective repulsion, leading to a higher apparent Θ-point in two dimensions.

**Figure 2:**
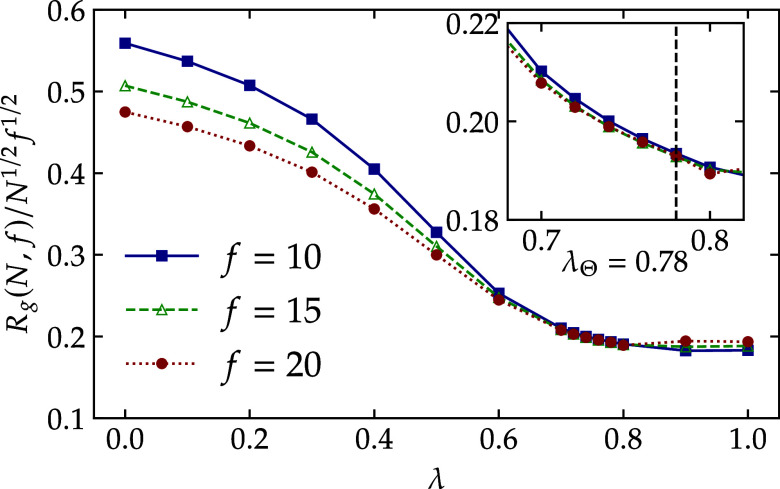
Radius of gyration normalized by the scaling law of Θ temperature plotted versus λ, all the curves passes a common point at λ_Θ_. The inset is a zoom in the region of the Θ-point, showing that all the curves are crossing near λ_Θ_ = 0.78. Data is produced from the simulation of single stars.

In Figure 3, we show monomer density profiles for our confined star polymers for different functionalities and solvent conditions resulting from simulations. For the athermal solvent case, λ = 0, shown in Figure 3a, our findings corroborate the claim that the confined stars behave as two-dimensional objects, as far as their scaling is concerned: indeed, the monomer profiles display a broad regime of a power-law dependence, ϕ(*f*, *s*) ∼ *s* ^–2/3^ with the two-dimensional exponent −2/3, cf. ϕ­(*f*, *s*) ∼ *s* ^–4/3^ in three dimensions. Similarly, the results for the Θ-condition, λ_Θ_ = 0.78, shown in Figure 3b, support once again both the two-dimensional character of the stars, corroborating the scaling ϕ(*f*, *s*) ∼ *s* ^0^, and the determination of the Θ point itself. Additional information that can be extracted from these profiles is the significant shrinking of the stars at the Θ-point in comparison to their size in athermal solvents as well as the fact that the *f*-stretching in Θ-conditions is much stronger than in athermal solvents, ∼*f* ^1/2^ vs ∼*f* ^1/4^, see eq 10.

**Figure 3:**
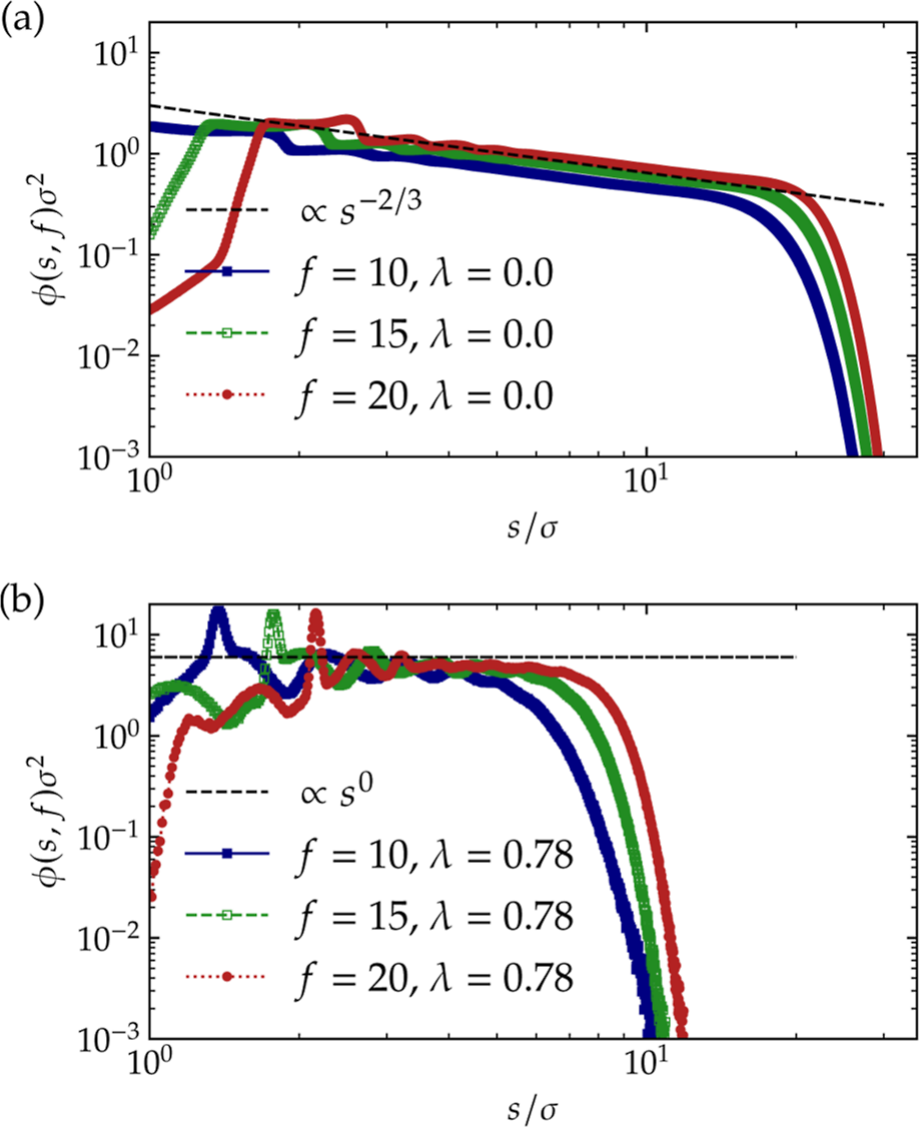
Monomer density ϕ­(*s*, *f*) of confined star polymers of functionality *f* as a function of the distance from the star center, *s*, for various values of *f* is simulated in our work. Panel (a) shows the density in athermal solvent conditions, λ = 0, and panel (b) in Θ-solvents, λ_Θ_ = 0.78. The dashed lines show the power-law dependencies according to eq 9, ϕ­(*s*, *f*) ∼ *s* ^–2/3^ and ϕ­(*s*, *f*) ∼ *s* ^0^, respectively. Data is produced from the simulation of single stars.

### 3.2 Effective Interactions: the Athermal Case

The effective force *F*_eff_(*d*) between two star polymers with their centers kept at separation *d* has been calculated by means of Molecular Dynamics (MD) simulations as explained in detail in the Supporting Information, and it is related to the effective interaction *V*_eff_(*d*) via the relation^7^ *F*_eff_(*d*) = −∂*V*_eff_(*d*)/∂*d*. Accordingly, we discuss below the effective interaction itself and we will assess the accuracy of the approximations developed for this quantity by comparing the opposite of its derivative with the force measured in the MD simulations.

We begin with the effective interaction for the case of athermal solvents, λ = 0, which we denote as *V*_0_(*d*). It has been shown based on field-theoretical arguments^28,29^ that for star–star separations *d* smaller than the star size, the effective interaction for athermal stars is logarithmically diverging, and it takes the form

11 $$\frac{V_{0}\left(d\right)}{k_{B}T}≅-\frac{2+9f^{2}}{24}\ln\left(\frac{d}{R}\right),,\left(d\leq R\right)$$

where *R* stands for a typical star size, e.g., the gyration radius or the corona radius of the polymer. The coefficient (2 + 9*f* ^2^)/24 is exact for all *f* ≥ 1, attaining the value (γ – 1)/ν = 11/24 for *f* = 1 and fulfilling the asymptotic scaling ∼*f* ^2^ for *f* ≫ 1. What is missing from eq 11 is the decay of the effective interaction for distances larger than the star size. It is understood that these are decaying exponentially fast with the interparticle separation^28^ and they have been previously modeled with a Gaussian potential^29^ for the case of adsorbed star polymers. For this study, we have found that the details of the tail of the interaction are crucial for reproducing accurately the correlation functions of the fluid, especially at low densities. This observation complements the previously mentioned fact that these tails influence the phase behavior of the system drastically as well.^30^ To allow for a more precise description of the potential tail, we introduce a single additional fit parameter α in the modeling of the interaction tail, namely

12 $$\frac{V_{0}\left(X\right)}{k_{B}T}=\frac{2+9f^{2}}{24}\{\begin{array}{l} -\ln X+\alpha ,,\text{if}\;X<1,\\ \alpha \exp\left[\left(1-X^{2}\right)/\left(2\alpha \right)\right],,otherwise, \end{array}$$

where X = *d*/*R*_g_ stands for the scaled distance of two alike star polymers, and α = α­(*f*) enters the expression in such a way that both the potential and its derivative are continuous at *X* = 1. As all variables are expressed in terms of their natural units, we expect that α­(*f*) will be of order unity.

At large separations, the effective force between the confined star polymers is weak and fluctuates strongly in simulations, so that it is inaccurate to attempt to determine the precise value of α by fitting the decay of the forces, as can be seen in Figure 4 for *f* = 20, similar plot is available in Supporting Information for *f* = 10, 15. Indeed, a few different values of α all lead to a similar quality of the fit of the tail of the interaction, offering therefore no conclusive evidence regarding the choice to be made. At the same time, the influence the tail has on the correlation functions is quite strong, especially at low- to intermediate concentrations, where the particles explore this domain of the interaction range. For these reasons, we resort to an approach for the determination of the decay parameter α that is based on the quality of the resulting radial distribution function *g*(*r*). According to Henderson’s theorem, if a monomer-solved system and a coarse-grained system produce identical *g*(*r*), their effective pair interaction differs only by an additive constant.^51^ Consequently, by comparing the radial distribution functions, we can confirm the correctness of the calculated effective pair interactions for star polymers and choose thereby the appropriate value of α.

**Figure 4:**
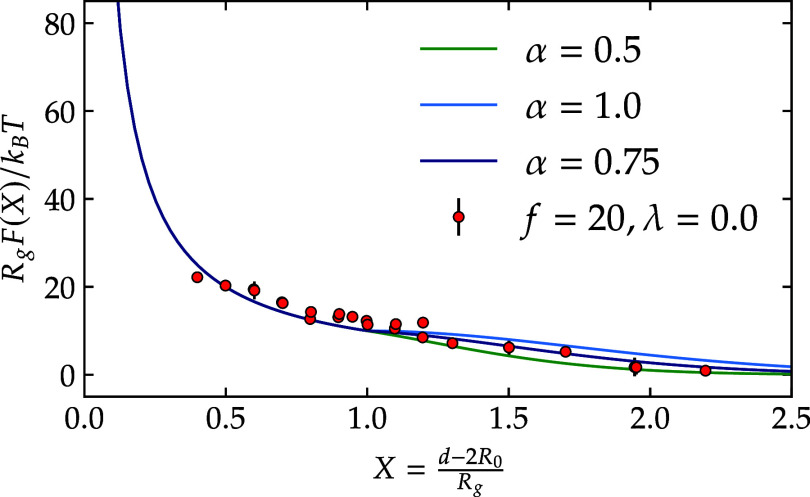
Relationship between normalized force and normalized core-to-core distance in athermal star polymers is presented herein. *R*_g_ represents the gyration radius of the polymers, and *R*_0_ is the core size of the stars, *d* is the core-to-core distance between two-star polymers. The data obtained from molecular dynamics (MD) simulations are shown as closed circles, and the curves illustrate the resulting fits based on eq 12 for various values of α.

To this end, we have simulated a total of 32 monomer-resolved star polymers of different functionalities for different concentrations ϱ, defined in eq 3 in Methods, i.e., ϱ ∈ [0.05, 0.15, ..., 0.25, 0.30], calculating the resulting radial distribution functions *g*_MR_(*r*). In addition, we evaluated the results of coarse-grained simulations involving 1024 particles using the effective interaction shown in eqs 12 and 15 for different values of α, obtaining thus the coarse-grained radial distribution functions *g*_CG_(*r*; α), depending parametrically on α, and calculated the root-mean-square error R(α) using the following expression

13 $$R\left(\alpha \right)={\left[\frac{1}{\left(r_{\max}-r_{\min}\right)}\int_{r_{\min}}^{r_{\max}}dr{\left[g_{MR}\left(r\right)-g_{CG}\left(r;\alpha \right)\right]}^{2}\right]}^{1/2}$$

where *r*_min_ = 0 and *r*_max_ = *L*/2, with the simulation box size *L*.

Figure 5a shows the quantity R(α) , calculated at low star density, ϱ = 0.05, for three different star functionalities. There is a single value of α for which a minimum occurs for each functionality and indeed all such values are of order unity, as anticipated, with a weak *f*-dependence. Moreover, the optimal value is decreasing with *f* monotonically, a feature in agreement with the fact that α^1/2^ represents a decay length of the monomer profile around the star center, which is indeed diminishing as *f* grows, see Figure 3a. Figure 5b–d show the effect of the parameter α on the accuracy of the coarse-grained prediction for *g*(*r*) in comparison with the true result obtained from the microscopic simulation and demonstrate that small changes in α have dramatic effects, in particular at low densities. In particular, values of α that are too low bring about both overpenetration of stars at low distances and a weaker correlations at large distances, a feature arising from the fact that a lower α implies a shorter-range and less steep potential in the region *x* > 1, see eq 12. At low concentrations, the particles find themselves at typical distances for which they feel precisely the potential tail, therefore the effect of α is particularly strong there. On the contrary, as can be seen in Figure 5d, as concentration grows the radial distribution function becomes less sensitive in the value of α, as now the particles feel mostly the logarithmic core of the effective interaction in the region *X* ≤ 1 of eq 12, for which α is merely an additive constant. In terms of the validity of the effective pair potential, the effective interaction of eq 12 is capable of accurately describing the correlations of the system all the way into the semidilute regime without the use of density-dependent parameters: indeed, the exact value of α can be employed at all concentrations, for any fixed choice of the functionality *f* and λ of the confined stars. It is noteworthy that the density dependence of α can be disregarded for two reasons: first, the representation of pair interactions in the radial distribution function remains valid at low densities, where many-body interactions are negligible; and second, our findings suggest that the influence of α diminishes at higher densities.

**Figure 5:**
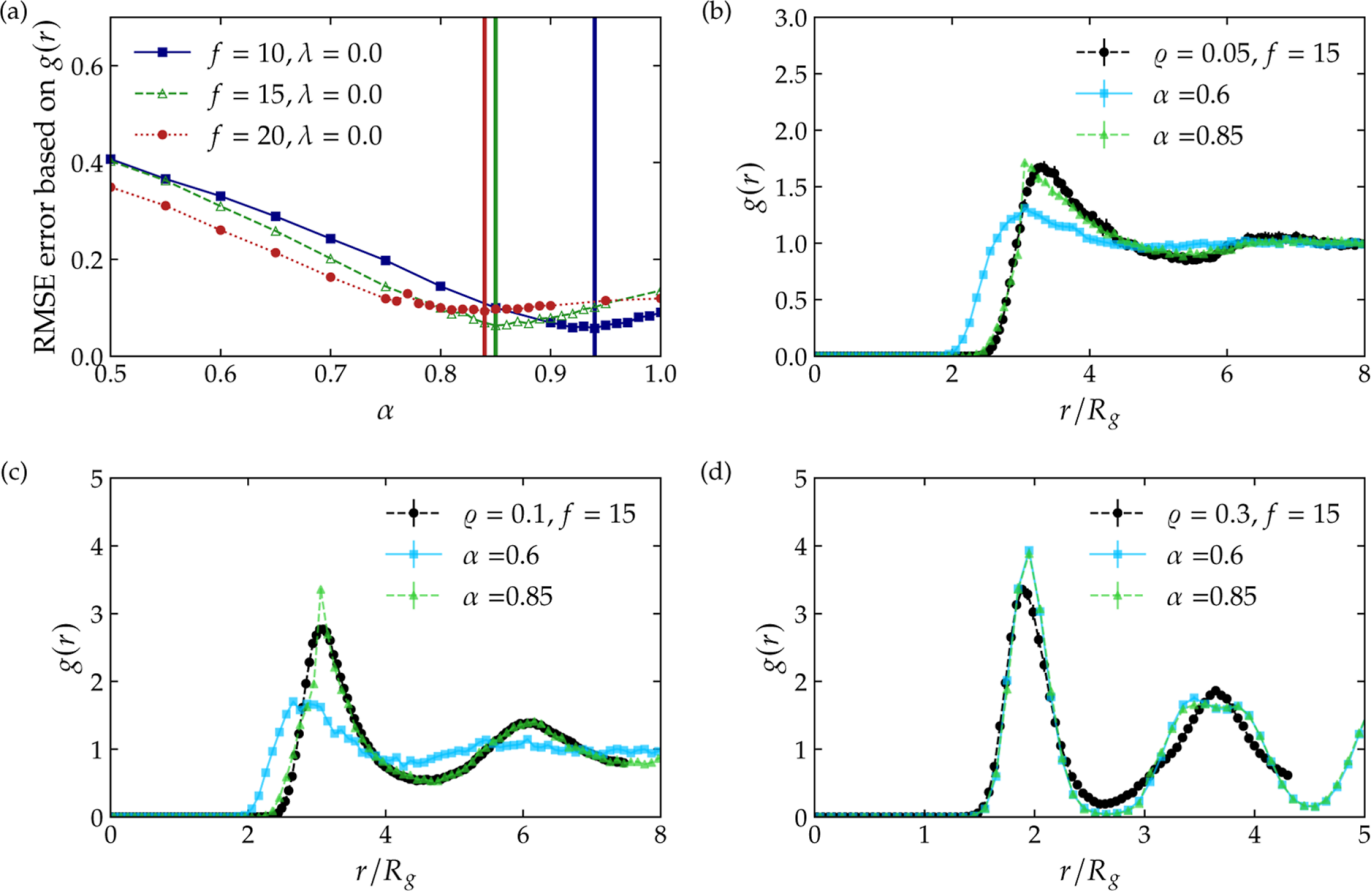
Dependence of the coarse-grained prediction for the radial distribution function *g*(*r*) against the monomer-resolved result for various concentrations ϱ and functionalities *f* of slit-confined, athermal star polymer solutions. Panel (a) shows the root-mean-square error R(α) the Root-mean-squared error, eq 13, as a function of α, calculated at concentration ϱ = 0.05, for three different star functionalities indicated in the legend. The vertical solid lines show the selected α, based on the least error. Panels (b–d) show examples of *g*(*r*) for different values of α in dilute and semidilute solutions, as indicated in the legends, for *f* = 15. The black symbols represent the monomer-resolved radial distribution function, the colored symbols are obtained in a coarse-grained fashion for different choices of α, are compared; the selected value, based on minimizing R(α) for *f* = 15, is α = 0.85, denoted by the green points. Lowering or increasing the α can largely influence the collective behavior of the system, especially in dilute solutions. In this figure, all results pertain to the athermal case, λ = 0. It is practical to see Figure 4 to see α affect the form of the potential weakly.

### 3.3 Effective Interactions: Effects of Solvent Quality

We now turn our attention to the case of good but thermal solvents, 0 < λ < λ_Θ_. As the conformations of the polymeric arms are still governed by the statistics of the self-avoiding walk, an effective entropic repulsion of the form of eq 12 will still be valid, albeit with a λ-dependent parameter α present in this case. The assertion that α will attain a λ-dependence arises from the fact that α^1/2^ is a decay length and the overall star conformation becomes tighter as λ grows; thus we expect α­(λ) to be a monotonically decreasing function, as we will shortly confirm. In addition, trivially, the radius of gyration *R*_g_ decreases with the worsening of the solvent quality; see Figure 2.

In addition to entropic repulsion, an effective attraction arises from the enthalpic interaction when the arms of a one-star polymer come closer to one another or to the same star polymer, compared to the isolated case. We thus split the effective potential *V*_eff_(*d*; λ) in the form

14 $$V_{eff}\left(d;\lambda \right)=V_{0}\left(d,\lambda \right)+V_{att}\left(d;\lambda \right)$$

where *V*_0_(*d*, λ) is given by eq 12 with an additional dependence α­(λ) and *V*_att_(*d*; λ) is the term arising from the attractions.

The functional form of such an attraction is difficult to estimate because the polymer shape is dependent on the solvent quality and the distance of the other star; associated with this issue is the question of whether two approaching stars will display overlap or faceting as they get close to each other. On the other hand, we know that the attractive term can be scaled by *f* ^2^, as the number of interacting arms of the two star polymers, and λ, the strength of attraction potential. Therefore, the solvent-mediated attraction has the form *V*_att_(*d*; λ) = −*f* ^2^λΦ­(*d*/*R*_g_; λ), with some function Φ­(*X*; λ). We found that an exponentially decaying function provided an accurate estimate of this functional form, i.e.

15 $$\Phi \left(X;\lambda \right)=A_{0}\left(\lambda \right)\exp\left[-\kappa \left(\lambda \right)X\right]$$

with the additional fit parameters *A*_0_(λ) and κ­(λ).

The fit parameters κ­(λ) and *A*_0_(λ) have been determined by fitting the effective forces resulting from simulations at the close-distance range, *X* ≤ 1, at different λ-values, and they are shown in the inset of Figure 6c. The parameter α­(λ) has been determined, as for the case λ = 0, by minimizing the mismatch between the monomer-resolved and coarse-grained radial distribution functions *g*_MR_(*r*) and *g*_CG_(*r*), respectively, details are shown in the Supporting Information. The overall results are illustrated in Figure 6 for three cases, demonstrating a good fit. Furthermore, the results for α­(λ) are shown in Figure 7. A decrease in solvent quality reduces the size of the decay region for the monomer density around a star corona, leading to a decrease in α, with λ. We emphasize that the obtained results hold for thermal but still good solvents, 0 ≤ λ < λ_Θ_; additional information regarding the star’s conformation for poor solvents, λ > λ_Θ_ can be found in the Supporting Information.

**Figure 6:**
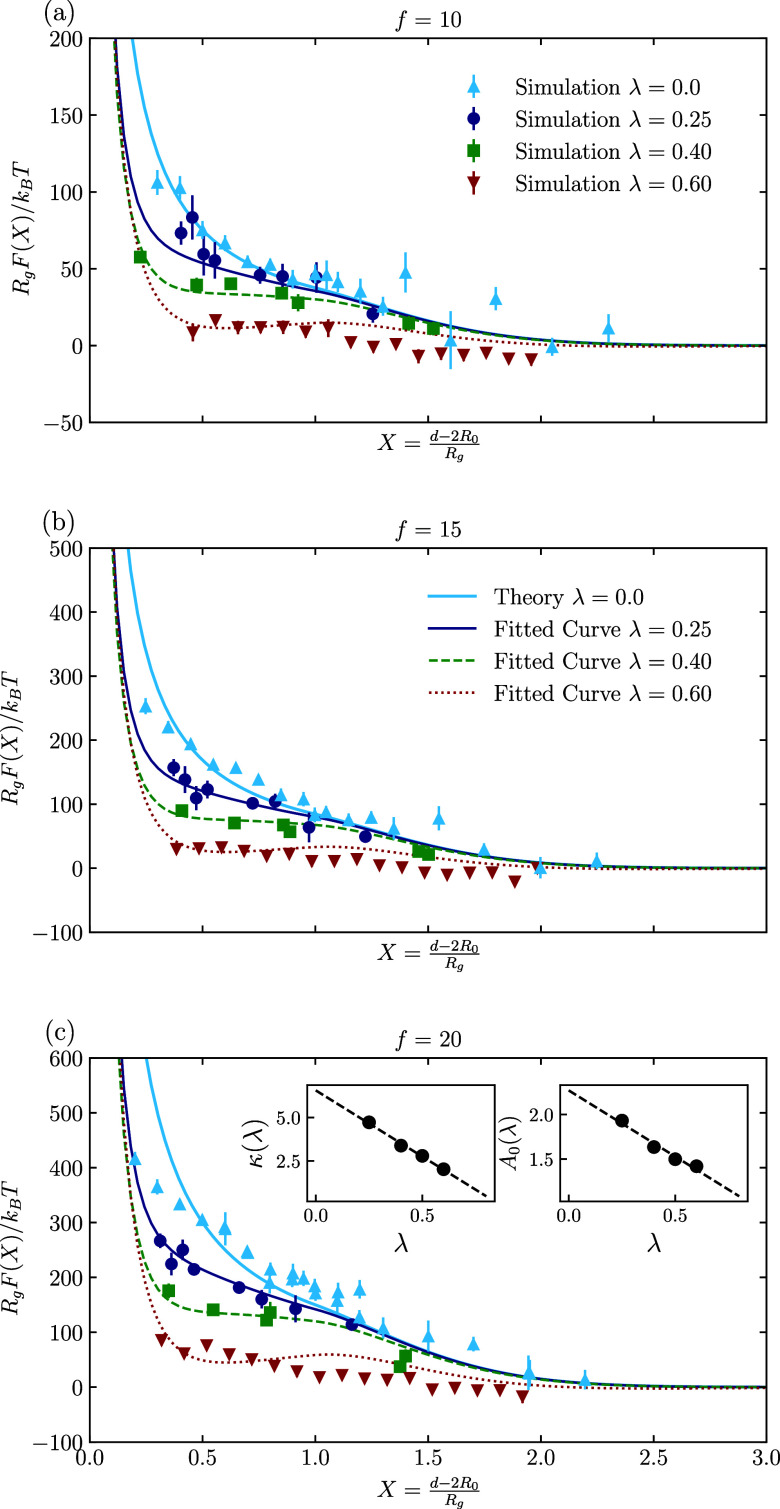
Scaled effective force between star polymers for the athermal case and three selected good solvent qualities, against the scaled distance between two stars, *R*_g_ represents the gyration radius of the polymers, and *R*_0_ is the core size of the stars, *d* is the core-to-core distance between two-star polymers. Subfigure (a) shows the interaction between stars with *f* = 10 arms, and (b,c) for *f* = 15 and *f* = 20, respectively. In all three subplots, sky and blue are for the thermal case, and navy blue, green, and dark red represent the interaction for λ = 0.25, λ = 0.4, and λ = 0.6, respectively. Inset plots in subfigure (c) show the fit for the *A*_0_ and κ in eq 15. They both show linear behavior.

**Figure 7:**
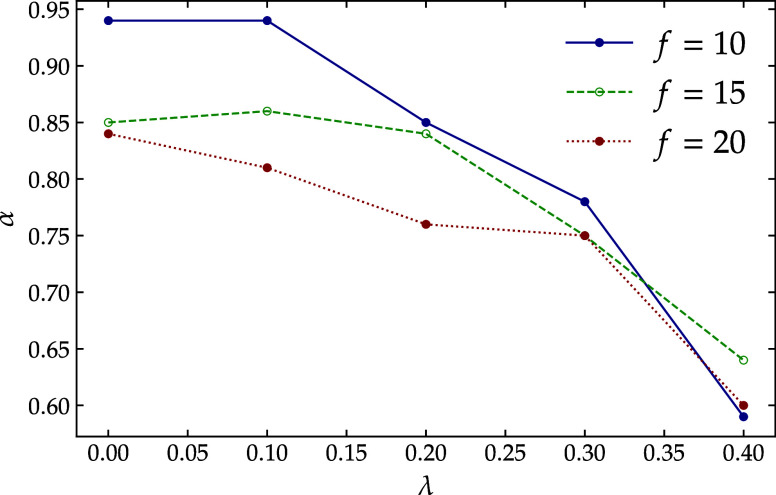
α parameter as a function of λ. The blue line represents systems with 10 arms, green with 15 arms, and red with 20-arm star polymer. By decreasing solvent quality and increasing the functionality, the parameter α decreases.

### 3.4 Morphological Coarse-Graining

Due to the soft pair interactions between star polymers, these molecules can form dense solutions with packing fractions significantly exceeding those in closed-packed systems. This behavior arises from the system’s response at the individual soft colloid level. Polymers have the ability to shrink, deform, and interpenetrate each other, leading to solutions where the interpolymer distance is less than the size of the unperturbed polymers.^31,34^ While this aspect is intriguing, the response of a single particle ultimately influences the material properties at the bulk level. Consequently, the behavior of these systems surpasses the understanding provided by simple point particle models with pair potentials. Such models fail to address many critical issues in soft matter, including crystallization, glassy behavior, jamming, and materials’ rheological and mechanical responses.^22,32^ To overcome the shortage of this model, a more complex, geometric model that is able to address the shape response of single molecules needs to be developed. This section aims to investigate our two-dimensional monomer-resolved dense solution of star polymers, which consists of two distinct domains.

As previously noted, the monomer density of the star polymer includes both a core and a tail component, see Figure 3. By incorporating and integrating methodologies used for microgels and polymer-grafted nanoparticles, we endeavor to explore the phenomena of shrinkage, deformation, and interpenetration in another soft particle prototype, star polymers, in a two-dimensional context. The two-layer model introduced in ref 33 will serve as the foundation for our study. This simple model, originally designed for spherical nanoparticles grafted with polymer brushes, decomposes the brush thickness into two distinct layers. One layer consists of stretched polymers that are not invaded by the chains from neighboring nanoparticles, while the other layer comprises unperturbed chains that are shared among various nanoparticles. We will extend this model to two-dimensional star polymers, incorporating insights previously applied to microgels. In this context, these dense soft colloids can be divided into two parts: a core that remains stable in its colloidal shape and is encapsulated within a soft, fuzzy layer. We will analytically derive the polymer size, area, and overlap, and compare these findings with simulation results.

We begin our analysis by defining the overcrowding parameter as described by Midya et al.^33^ This parameter, denoted as *x*, quantifies the overcrowding and extension of polymer chains by looking at the ratio of unperturbed chains that occupy the space of a star in a melt of linear polymers with identical monomer density and star functionality (referring to the chains that occupy the grafted chain melt). In a two-dimensional framework, considering the total star size *R*_tot_, the number of chains in the unperturbed melt can be expressed as *πρ*_m_ *R*_tot_^2^/*N*, where ρ_m_ = ϱ*Nf*/*R*_g_^2^ represents the monomer density within the system. Since we are operating within a melt, we can assume the chains to be Gaussian, leading to the relation *R*_tot_^2^ = *b* ^2^ *N*, where *b* = 1.5σ is defined as the Kuhn length. Thus, the overcrowding parameter can be formulated as follows

16 $$x=\frac{f}{\pi b^{2}\rho_{m}}$$

Utilizing the analytical two-layer model, we treat our system as being in a melt condition and assume a uniform distribution of monomer density, irrespective of the specific star polymer from which the monomers originate. As we increase the density of the star polymer solution, we observe a consistent distribution of monomers, independent of the number of arms or the solvent quality. In dense solutions, all star polymers will shrink, leading to a monomer density comparable to that at Θ temperature, as illustrated in Figure 3b. We present the monomer density profile in melt configuration in Figure 8. In this framework, ρ_s_ represents the monomers associated with the chains originating from the central core, while ρ_o_ denotes the monomers from adjacent star polymer chains. The parameter ρ_m_ indicates the local monomer density. The interpenetration layer width is referred to as *h*_inter_, while the intact central region, free from interpenetration, is designated as *R*_d_. This central region comprises the core with a radius *R*, in addition to the dry segments of the chains represented by *h*_dry_. The total star radius is defined as *R*_tot_ = *h*_dry_ + *h*_inter_/2, and can be thought as average interparticle distance. To delineate these distinct sections, we employed the product ρ_s_(*r*)­ρ_o_(*r*), in accordance with the methodology established by Midya et al.^33^

**Figure 8:**
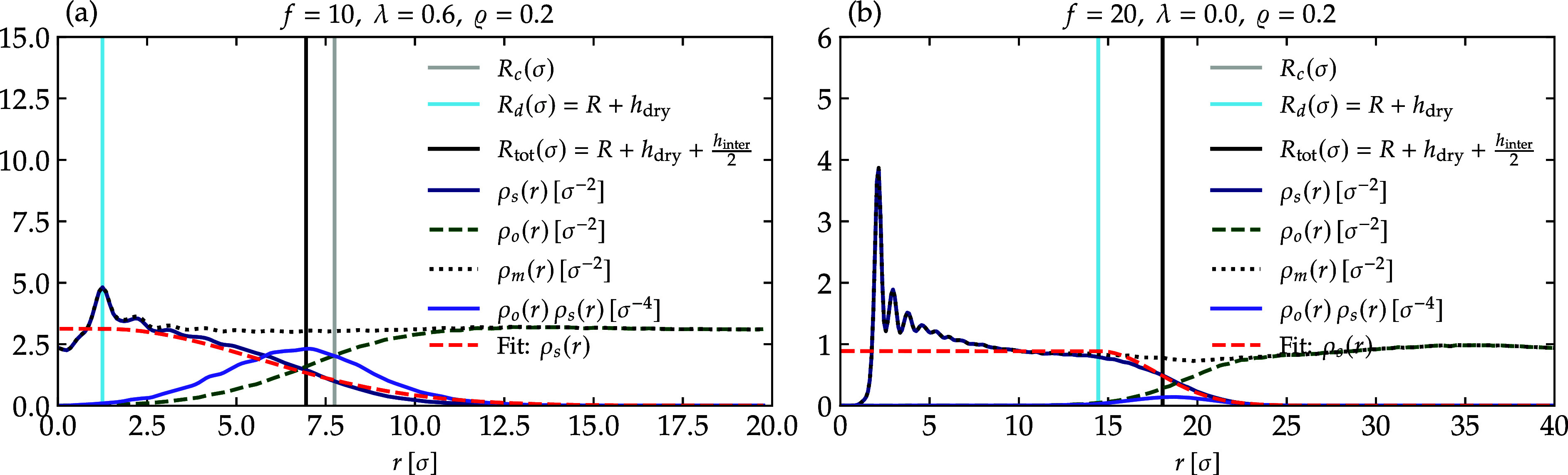
Various monomer density profiles of star polymers surrounding the central core in dense solutions, where the density ϱ = 0.2, as indicated in the legends. Panel (a) illustrates the results for *f* = 10 and λ = 0.6, while panel (b) reflects similar findings for *f* = 20 in an athermal solution. In this figure, we denote by ρ_m_(*r*) the local monomer density, whereas in the text ρ_m_ is a global quantity. Note that indeed, away from the center of the reference star, ρ_m_(*r*) is indeed rapidly converging to its bulk value ρ_m_.

The density profile of stars can be described by the following expression

17 $$\rho_{s}\left(r\right)=\{\begin{array}{l} \rho_{m},,r\leq R_{d}=R+h_{dry},\\ \rho_{m}\exp\left[-\frac{{\left(r-R_{d}\right)}^{2}}{2\sigma_{shell}^{2}}\right],,otherwise. \end{array}$$

This estimated profile will allow us to derive analytical expressions for *h*_inter_, *R*_d_, and σ_shell_. We begin with the space-filling assumption

18 $$\pi \rho_{m}\left[{\left(R_{d}+\frac{h_{inter}}{2}\right)}^{2}-R_{d}^{2}\right]=fn_{inter}$$

where *n*_inter_ denotes the number of monomers in the interpenetration layer. Moreover, based on the Gaussian distribution of chains in the melt, we can relate *h*_inter_ to the number of monomers through the equation $h_{inter}=bn_{inter}^{1/2}$ . This space-filling assumption yields an expression akin to that of grafted nanoparticles in three-dimensional space^33^

19 $$\frac{h_{inter}}{R_{tot}}=\frac{1}{x+\frac{1}{4}}$$

Additionally, using eq 17, we derive an implicit equation for σ_shell_

20 $$2\pi \rho_{m}\int_{R_{d}}^{R_{d}+h_{inter}}\exp\left[-\frac{{\left(r-R_{d}\right)}^{2}}{2\sigma_{shell}^{2}}\right]rdr=fn_{inter}$$

Consequently, one can predict the length scales *R*_d_, *h*_inter_, and σ_shell_ solely using the overcrowding parameter *x* and the average interparticle distance *R*_tot_. Figure 9 shows a comparison of the theoretical prediction of the two-layer model and the corresponding simulation results for *h*_inter_, and σ_shell_.

**Figure 9:**
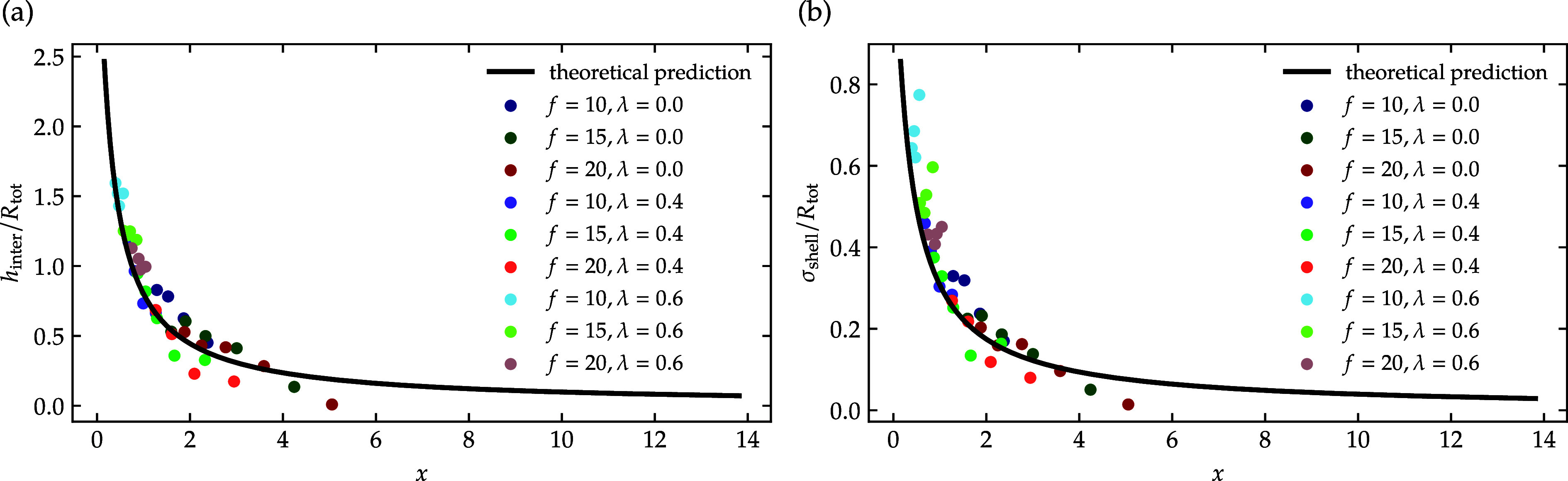
Comparison of theoretical two-layer model predictions (black solid line) and simulation results (scattered) for (a) the normalized interpenetration-layer thickness *h*_inter_/*R*_tot_ and (b) the normalized Gaussian shell width σ_shell_/*R*_tot_ as functions of the overcrowding parameter *x*. Data are shown for star polymers with functionality *f* = 10, 15, 20 in athermal (λ = 0), and good solvent conditions (λ = 0.4, λ = 0.6).

The star polymers extend throughout the entire dry section and partially into the interpenetration layer. In areas where they overlap with the interpenetration layer—though not completely or only in half—they exhibit a fuzzy appearance, potentially covering the layer fully in some directions while only partially in others. Furthermore, the interpenetration of star polymers is significantly more complex than what is outlined by the distribution of monomer density among the stars and other components. Consequently, estimating the density of star polymers, as expressed in eq 17, can prove more advantageous for determining the area occupied by the star polymers and the regions of overlap. Building on this understanding, we will define additional parameters: *R*_f_, *R*_c_, and *R*_s_. These parameters, derived from the study of microgel faceting and interpenetration, are defined as follows. First, *R*_f_ = *R*_tot_ + *h*_inter_, representing the full extent of the star polymers. Second, *R*_c_ expresses the size of the core section, which we expect the star polymer to cover in all spatial directions, irrespective of faceting. Therefore

21 $$R_{c}=\{\begin{array}{l} R_{d},,\text{if}\;\rho_{m}\left(r\right)\leq 1,\\ the\;distance\;r\;at\;which\;\rho_{s}\left(r\right)=1,,otherwise. \end{array}$$

Figure 8a illustrates a scenario with a very high monomer concentration, where *R*_c_ exceeds *R*_d_ and even the total star polymer size, denoted as *R*_tot_. In contrast, Figure 8b displays a situation with a monomer density lower than one, revealing that *R*_c_ is obscured by *R*_d_ and is thus not visible. Additionally, the equation $R_{s}=\frac{\left(R_{f}-R_{c}\right)}{2}$ defines half of the soft shell, which encompasses a region with a high probability of locating monomers that are distinct from the dangling chains found in the outer region of star polymers.

Figure 10 depicts a star polymer alongside its surrounding surface mesh, the latter drawn using different criteria regarding the local monomer density. In panel (a), the mesh encompasses all the monomers of the star polymer, whereas panels (b) and (c) illustrate the removal of monomers from the outer region and from within the soft shell, respectively, for clarity. When comparing these constructed surface meshes, it becomes apparent that including all monomers in surface mesh calculations results in an overestimation, as the surface mesh will extend over a large empty area. Moreover, the dangling chains are primarily small fluctuations on the surface of these soft colloids, and, crucially, they are not detectable in experiments, leaving their role inadequately understood.^31,34^ Conversely, entirely removing the soft shell, as shown in panel (c), results in an underestimation of the star polymer surface. Construction of the mesh based entirely on the core region, *r* > *R*_c_, downgrades the star polymers to simple colloids, disregarding their intrinsic nature as soft, deformable entities. Consequently, the optimal continuous model for describing them is a surface mesh constructed from the monomers in the core section, along with them in half of the soft fuzzy part, i.e., choosing the distance *R*_c_ + *R*_s_ from their center as the cutoff to construce the mesh, as shown in Figure 10b.

**Figure 10:**
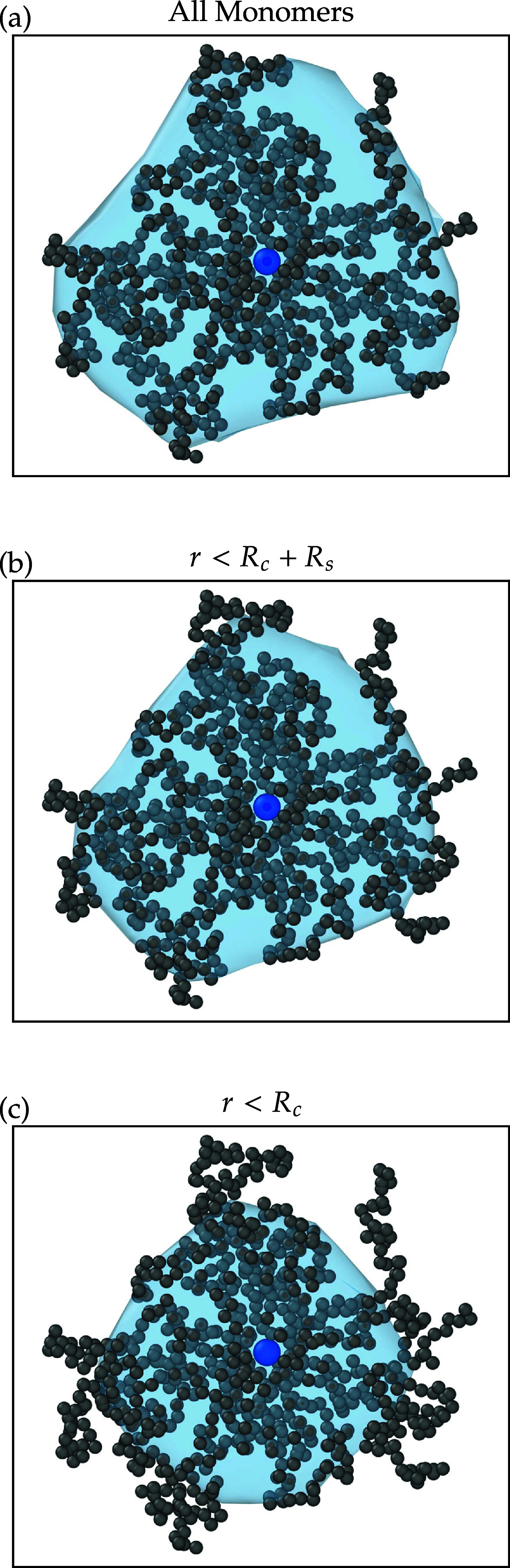
Visualization of a single slit-confined star polymer with functionality *f* = 15, solvent parameter λ = 0.4, monomer density ρ_m_σ^2^ = 1.43 captured from simulation snapshots. (a) Surface mesh covering all monomers. (b) Mesh after removing monomers in the outer (dangling-chain) region. (c) Mesh showing only the core monomers within the dry section (i.e., excluding the soft shell).

The first step in examining the evolution of the internal structure of star polymers involves analyzing their size as a function of concentration. Figure 11 illustrates the size of star polymers in relation to monomer density, presented in four different ways: Figure 11a shows the radius of gyration, while Figure 11b displays the total span of the polymer, denoted as *R*_f_. Figure 11c,d depict the core section and the width of the soft shell, respectively.

**Figure 11:**
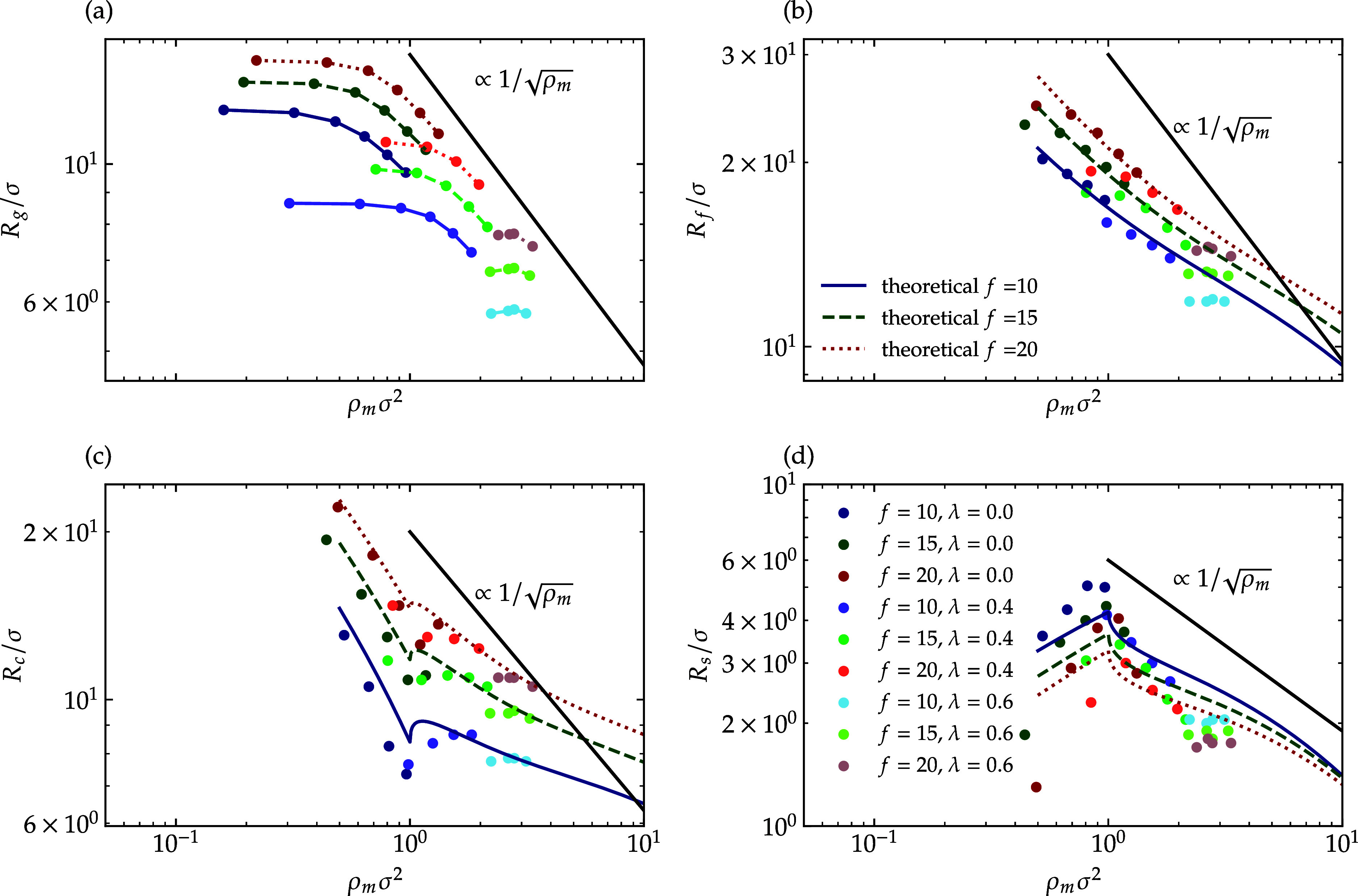
Size of slit-confined star polymers as a function of monomer density ρ_m_. Panel (a) shows the radius of gyration *R*_g_, panel (b) the total span *R*_f_, panel (c) the core radius *R*_c_, and panel (d) the soft shell width *R*_s_. Data are plotted for functionalities *f* = 10, 15, 20 and solvent parameters λ = 0.0, 0.4, 0.6, demonstrating isotropic shrinking ∼ρ_m_^–1/2^ at high densities. Symbols denote MD simulation results. Solid lines in subfigures (b–d) show analytical two-layer model predictions. The blue solid line represents *f* = 10, the green dashed line represents *f* = 15, and the red dotted lines represent *f* = 20 in all subfigures.

According to polymer theory, a two-dimensional polymer will exhibit isotropic shrinking behavior of approximately ∼ρ_m_^–1/2^, as derived in the Supporting Information. This trend is indeed observed when examining the radius of gyration as a function of monomer density. Regardless of the values of *f* and λ, all star polymers share the same scaling behavior at high concentrations. At sufficiently high concentrations, star polymers with similar *f* values converge onto the same curve, as they all assume a collapsed form. In contrast, at lower concentrations, they exhibit no significant shrinking and demonstrate varying onsets for shrinking. This variation arises from their different unperturbed sizes, resulting in contact at different monomer densities.

The three remaining values, *R*_f_, *R*_c_, and *R*_s_ are also possible to be obtained through the analytical two-layer model. Therefore, they are also plotted to show the compatibility with the simulation results. The term *R*_f_ can be expressed as *h*_inter_ + *R*_tot_ and can be calculated using only the overcrowding parameter. Although *R*_c_ and *R*_s_ can be derived easily through eq 17, they require one additional parameter for estimation, and the overcrowding parameter is not enough. The results are plotted in Figure 11; in deriving the analytical expressions for these quantities, which are displayed with the continuous curves in Figure 11b–d, we set, and for simplicity, $R_{tot}=\sqrt{Nf/\pi \rho_{m}}$ in the calculations.

The total span of the polymer decreases at a rate less than ρ_m_^–1/2^ (see Figure 11 panel (b)). This behavior is indeed a result of the growth of the interpenetration layer with increasing density. This phenomenon is also evident when observing the core size in Figure 11 panel (c). Initially, the core size decreases significantly, indicating a reduction in the dry section as predicted. However, once the monomer density reaches the threshold value, ρ_m_σ^2^ = 1, the shrinkage becomes less pronounced. This change reflects the increase in the interpenetration width (*h*_inter_) and consequently of the width σ_shell_ in eq 17, as the solution density rises. Similarly, at low concentration, the width of the soft shell begins to increase, indicating a growing interpenetration layer. However, upon reaching a density of 1/σ^2^, it experiences a marked reduction (more pronounced than ρ_m_^–1/2^). This behavior at high concentrations aligns with the findings observed in the microgels.^34^

To examine the overall shape deformation and overlapping of star polymers, we calculate their average area, which can be derived analytically as follows

22 $$Area=\pi R_{c}^{2}+2\pi \int_{R_{c}}^{\Lambda }\rho_{s}\left(r\right)rdr$$

where the Λ is chosen to exclude contributions from the outer or soft region of the monomers. Note that eq 22 incorporates an implicit assumption that the stars are essentially isotropic, at least on average; if, e.g., the stars have polygonal shapes, as is the case for strong faceting, it will not properly capture their shrinkage as ρ_m_ grows. To test its validity, we also performed numerical calculations by projecting the surface mesh onto the *x*–*y* plane in addition to the analytical approach.

Results from theory and simulation are shown Figure 12, which illustrates the area under three distinct scenarios: panel (a) depicts the area encompassing the entire monomer profile; panel (b) excludes the dangling chains, *r* < *R*_c_ + *R*_s_; and panel (c) concentrates exclusively on the core monomers, *r* < *R*_c_. In both the numerical and the theoretical assessments, the quality of the solvent does not impact the area occupied by star polymers: indeed, a single curve fits all λ-values for a given *f*. Instead, the space occupied by the polymer is dictated solely by its functionality, which directly affects the degree of polymer softness and the density of the monomers. We also note that our findings diverge from theoretical predictions when we exclude the dangling chains or the softer regions, see Figure 12b,c. This discrepancy disappears when all monomers are included in the area calculation, as shown in Figure 12a. Furthermore, as illustrated in Figure 11, the differences in parameter calculations are less pronounced than in the area assessment. This variation stems from faceting and shape deformation. While theoretical models assume angular symmetry and average circular shapes, star polymers tend to deform to fit within their Voronoi cells under dense conditions. This behavior is not fully captured in analyses that consider all monomers, as the fluctuations associated with the dangling chains can occupy a substantial volume without contributing to surface coverage. On the contrary, when considering the inner parts of the stars, the deviations from circularity are more persistent, similar and reproducible between different stars, so that they survive the averaging over all stars and cause deviations from the analytical results derived on the basis of eq 22, which is based on the assumption of circular symmetry.

**Figure 12:**
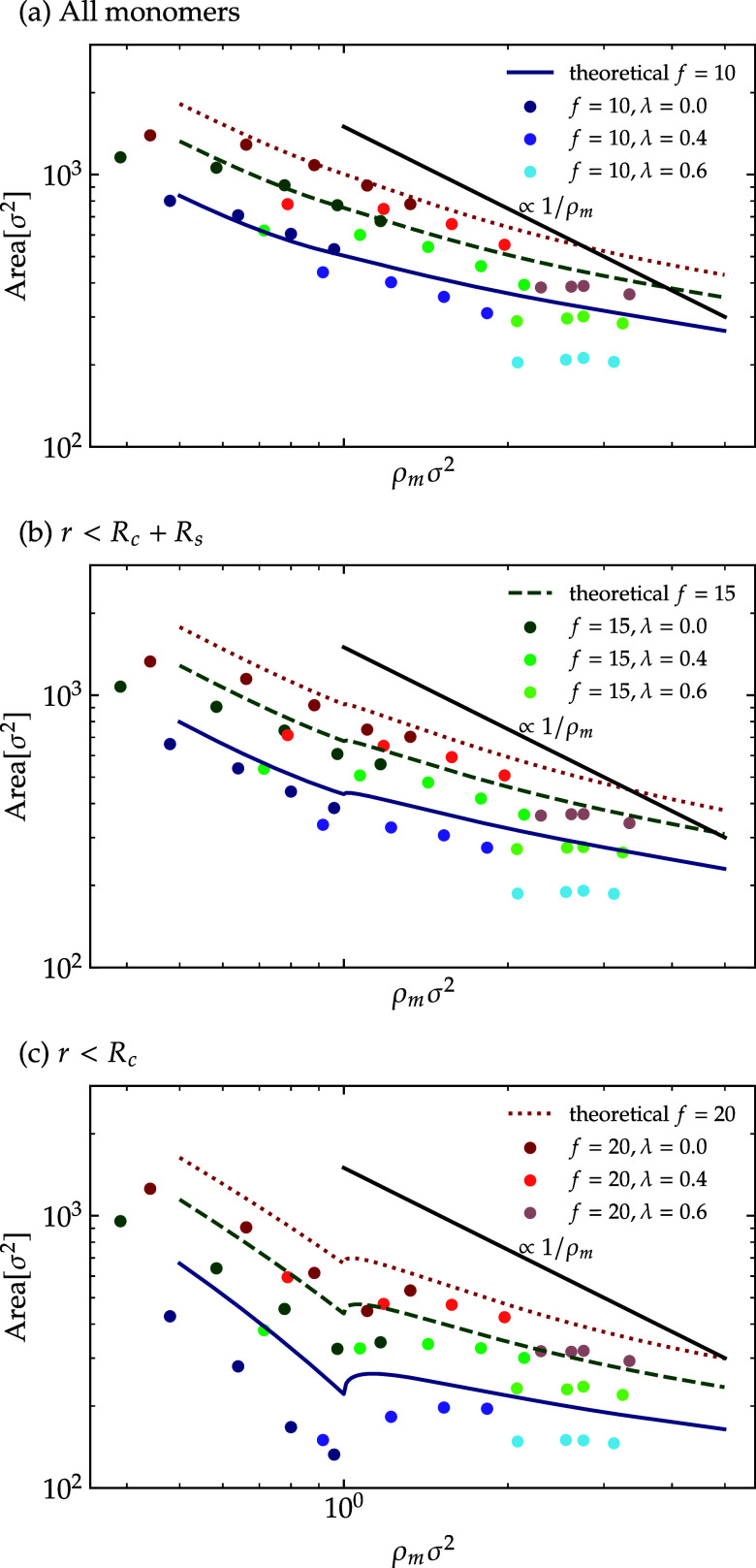
Projected polymer area under three scenarios: (a) including all monomers; (b) excluding outer dangling chains; (c) including only core monomers. Data are shown for star polymers with functionalities *f* = 10, 15, 20 and solvent qualities λ = 0.0, 0.4, 0.6, compared against analytical two-layer model predictions (lines). The increased deviation upon removing dangling-chain contributions arises from polymer faceting and star deformation. The blue solid line represents *f* = 10, the green dashed line represents *f* = 15, and the red dotted lines represent *f* = 20 in all subfigures.

Once the area of the star polymers has been computed, determining the overlap becomes straightforward. By assuming that the stars are filling the space, we can logically conclude that as the solution’s density increases, we first observe overlap in the outer regions before it extends to the inner regions. Consequently, the overlap parameter ψ is defined as follows, for all monomers or by removing monomers in the outer region

23 $$\psi =\left\langle \frac{\cup_{i>j=1}^{32}\left(A_{i}\cap A_{j}\right)}{\cup_{i=1}^{32}A_{i}}\right\rangle \approx \frac{\rho_{m}\cdot Area-Nf}{\rho_{m}\cdot Area}$$

where *A*_*i*_ refers to the instantaneous area the *i*th star polymer is encapsulated. The details of this mesh consideration for each star are explained in the Methods.

The results of this calculation are depicted in Figure 13a. It is clear that a reduced number of arms, which indicates a softer star polymer, leads to an increased overlap. Additionally, solvent quality appears to have minimal impact. Furthermore, it is essential to note that the calculations presented here exclude dangling chains, as this surface mesh effectively captures the fundamental characteristics of the polymer, as explained earlier. Similar plots, for other surface meshes, can be found in the Supporting Information.

**Figure 13:**
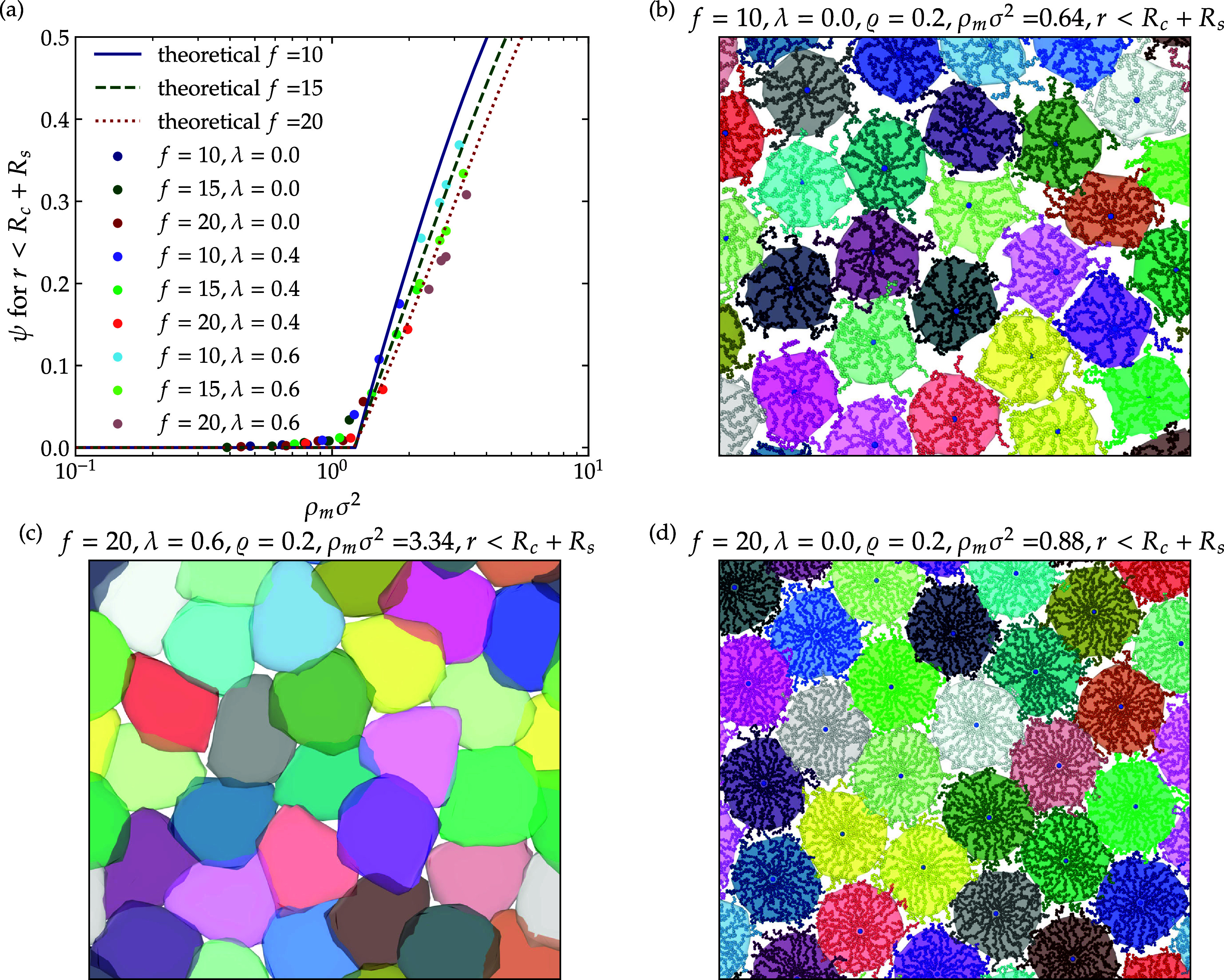
(a) Overlap parameter ψ computed analytically (lines) and measured from simulations (symbols) as a function of the monomer density. (b–d) Projected simulation snapshots of the polymer surface mesh illustrating overlap for representative state points at fixed star concentration ϱ = 0.2: (b) *f* = 10, λ = 0.0, (ρ_m_σ^2^ = 0.64); (c) *f* = 10, λ = 0.6, (ρ_m_σ^2^ = 3.13); (d) *f* = 20, λ = 0.0, (ρ_m_σ^2^ = 0.88).

While we employed a straightforward analogy to compute the overlap parameter analytically, our findings demonstrate a quantitatively good agreement with numerical results. Additionally, we have included three snapshots of star polymers featuring varying functionalities and solvent qualities. A comparison between Figure 13b,d highlights the impact of polymer softness. In Figure 13b, we observe how the softer polymers deform and exhibit variability, with dangling chains penetrating nearby surface meshes. Conversely, Figure 13d presents similar surfaces, almost resembling a hexagonal shape (their Voronoi cell). Furthermore, Figure 13c illustrates that, at high monomer densities, overlap becomes dominant. The faceting and shape deformation observed at lower concentrations are influenced by the neighboring star shapes, as seen in Figure 13b,d.

## 4 Conclusions

We have put forward two complementary approaches for coarse-graining soft colloidal particles, in the form of star polymers, in quasi-two dimensions. In the first approach, which aims at the derivation of effective Hamiltonians in which only the star centers feature as degrees of freedom, all monomers are canonically traced out. We have thus derived effective interactions for a broad range of solvent quantities, ranging from the athermal to the Θ-solvent, a set of ultrasoft interaction potentials. The latter features a logarithmically divergent repulsion at small separations with a longer-range attraction at larger distances, the latter becoming stronger as the Θ-point is approached. Our approach yields analytical expressions for all values of the functionalities and the parameter λ describing the solvent quality considered, and the pair potential approximation is shown to be valid well into the semidilute regime, where phase transitions are expected. In particular, these interactions will be useful in analyzing both the liquid-hexatic-solid phase transition and the emerging liquid–gas transitions (for λ > 0) in such systems. Work along these lines is currently under way.

Although we demonstrated the effective pair potential approximation to be valid even in concentrated solutions, it is bound to break down as the density further grows, as it has been shown for e.g., star polymers in three dimensions.^52^ Accordingly, it is necessary to develop a more general, morphological model, which considers each star as an elastic, geometrical object incorporating surface tension or elasticity for colloids can effectively capture the faceting and interpenetration phenomena. This will simplify many calculations related to the collective behavior of dense soft colloid solutions.

We have thus further put forward a two-layer model for star polymers at high concentrations, which does not eliminate the monomeric degrees of freedom but instead preserves information about the star conformation, allowing us to also quantify interpenetration and faceting, which play a crucial role in determining dynamical and rheological properties of soft particle suspensions.^21,22,53,54^ The model was shown to be robust; by integrating a straightforward function for monomer density, we can predict features that are often challenging to capture in experimental settings. This model can provide insights, such as in a multimodal solution of star polymers with varying degrees of polymerization and functionality. It can determine the optimal ratios of these polymers to achieve a film with maximum overlap while minimizing weight. Such predictions are invaluable for designing coatings, resulting in durable, stretchable, and viscoelastic materials tailored to specific user requirements, and nanotechnological applications.

## Supplementary Material



## Data Availability

All data needed to evaluate the conclusions in the paper are present in the paper and/or the Supporting Information.

## Notes

A detailed version of the Method section. Detailed analysis of the star polymer shape as a function of the number of arms and solvent quality. Effective interaction calculations, showing details of tail parameter computations, mainly for thermal star polymers. Investigating the star overlap for different surfaces meshes (PDF)
