# Supplementary material for: Coarse-Graining of Slit-Confined Star Polymers in Solvents of Varying Quality
Source: Macromolecules. 2025 Oct 31;58(21):11827–40. doi: 10.1021/acs.macromol.5c01343 (PMC12613821; doi:10.1021/acs.macromol.5c01343)
Supplement: Supplementary file 1 [file ma5c01343_si_001.pdf]

Supporting Information: Coarse-graining of slit-confined star polymers in solvents of varying quality

Reyhaneh A. Farimani* and Christos N. Likos*

Faculty of Physics, University of Vienna, Boltzmanngasse 5, A-1090 Vienna, Austria.

E-mail: reyhaneh.afghahi.farimani@univie.ac.at; christos.likos@univie.ac.at

Computational Model

In our monomer-resolved simulations, we utilized a coarse-grained model that incorporates the varying quality of the solvent, as initially introduced by Huissmann et al.[1] Each star polymer consists of a single core with f arms, with each arm containing $n = 50$ monomers. To reduce the concentration of monomers at the center, we increased the core size linearly by $f/5$; therefore, the diameter of the cores of stars with 10 arms is 2σ, with 15 arms it is 3σ, and with 20 arms it is 4σ. Consequently, each star polymer contains $fn + 1$ monomers and fn bonds. The steric interactions between any two monomers at a relative distance r are modeled using the purely repulsive, truncated, and shifted Lennard-Jones potential (WCA),

$$V^{WCA}(r) = \begin{cases} 4\epsilon\left[\left(\frac{\sigma}{r}\right)^{12} - \left(\frac{\sigma}{r}\right)^{6} + \frac{1}{4}\right], & \text{if } r \leq 2^{1/6}\sigma, \\ 0, & \text{if } r > 2^{1/6}\sigma, \end{cases} \tag{1}$$

the solvent quality is modeled via an effective attraction between arm monomers as follows:

$$V^{att}(r,\lambda) = \lambda \begin{cases} -\epsilon, & \text{if } r \leq 2^{1/6}\sigma, \\ 4\epsilon\left[\left(\frac{\sigma}{r}\right)^{12} - \left(\frac{\sigma}{r}\right)^{6}\right], & \text{if } r > 2^{1/6}\sigma, \end{cases} \tag{2}$$

therefore, for $\lambda = 0.0$, we have only the WCA repulsive interaction (usual bead spring model), representing the athermal case, and by increasing λ, one is decreasing the solvent quality.

The bonded interaction is modeled via $FENE$ dumbbell model:

$$V^{FENE}(r) = \frac{1}{2}kR_0^2 \ln\left[1 - (\frac{r}{R_0})^2\right]. \tag{3}$$

In the equations 1 to 3, the variable r denotes the distance between monomers, while ϵ and σ serve as the reference scales for energy and length, respectively. The bead masses are utilized as the reference mass. The remaining parameters are fixed: $k = \frac{30\epsilon}{\sigma^2}$ and $R_0 = 1.5\sigma$. We have considered the cut-off for all the aforementioned potentials at $r_{cut} = 2.5\sigma$.

All star polymers are confined between two parallel, repulsive walls, resulting in a slit geometry. The interaction between the walls and the monomers is modeled using the Weeks-Chandler-Andersen (WCA) potential (equation 1), which operates exclusively in the $\hat{z}$ direction. The slit width is set to $H = 4\sigma$, and periodic boundary conditions are applied in the $\hat{x}$ and $\hat{y}$ directions, and the size in these directions was large enough to make sure no contact with the periodic image. A picture of a polymer between the two walls is represented in figure S1.

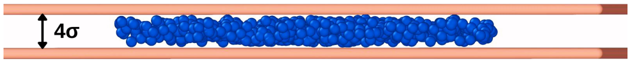

Figure S1: Snapshot of a star polymer in a slit.

The MD simulations were conducted for the monomer-resolved simulations. We solved the Langevin equation at a fixed temperature of $k_BT = 1.0\epsilon$ using the velocity Verlet

algorithm, implemented in the large-scale atomic/molecular massively parallel simulator (LAMMPS).[2]

$$m\ddot{\mathbf{r}}_n = \mathbf{F}_n - \gamma\dot{\mathbf{r}_n} + \mathbf{R}_n(t) \qquad n \in 1, 2, ..., Nf + 1 \tag{4}$$

In Equation 4, $\mathbf{r}_n$ represents the position of the nth bead, $\mathbf{F}_n$ denotes the deterministic forces acting on the nth bead, $-\gamma\dot{\mathbf{r}}_n$ represents the viscous forces, and $\mathbf{R}_n$ is a random Gaussian force representing the stochastic forces acting on the bead. The variables $\gamma = \frac{1}{4}\sigma/\sqrt{m\epsilon}$ and $\mathbf{R}_n$ are related through the fluctuation-dissipation theorem. The integration step is $\Delta t = 0.005\sqrt{m\sigma^2/\epsilon}$.

To directly assess the effective interaction between star polymers, we conducted simulations involving two star polymers with their cores positioned at $\mathbf{R}_1$ and $\mathbf{R}_2$. Initially, the distance between the cores is defined as $|\mathbf{R}_1 - \mathbf{R}_2| = D$, where D is significantly larger than the radius of gyration R_g (i.e., $D \gg R_g$). We allowed the system to equilibrate for $10^6\sqrt{m\sigma^2/\epsilon}$ time steps, followed by a further simulation of an additional $10^6\sqrt{m\sigma^2/\epsilon}$ time steps. After this, we gradually reduced the core-to-core distance by $\Delta D \leq 0.1R_g$ and permitted the system to equilibrate for another $10^6\sqrt{m\sigma^2/\epsilon}$ time steps before conducting another simulation for $10^6\sqrt{m\sigma^2/\epsilon}$ time steps. Throughout the simulation process, we gathered configuration data and calculated the forces as described below:

$$\begin{aligned}\mathbf{F}_i(D) = &- \nabla_{\mathbf{R}_i}\left(\sum_{j=1}^{2}\sum_{k=1}^{f}\sum_{l=1}^{n}\left(V^{WCA}(|\mathbf{r}_{kl} - \mathbf{R}_j|) + V^{att}(|\mathbf{r}_{kl} - \mathbf{R}_j|, \lambda)\right)\right)\\ &+ \sum_{k=1}^{f}\sum_{l=1}^{n} V^{FENE}(|\mathbf{r}_{kl} - \mathbf{R}_i|).\end{aligned} \tag{5}$$

We calculate the average force over time and across samples for each computed distance, denoted as $\langle\mathbf{F}_i(D)\rangle$, and then we repeat the procedure. A minimum of ten replicas were generated for each simulation.

For the simulations intended to measure the radial distribution, we considered a system of 32 star polymers located within a box of dimensions $(L_x, L_y, 4\sigma)$ with the old slit geometry,

with a length ratio of $L_x : L_y$ set at $2 : \sqrt{3}$. The concentration was defined as $\varrho = \frac{32R_g^2}{L_xL_y}$.

In establishing the initial configuration, we employed a Monte Carlo (MC) method with a parameter $\alpha = 0.5$ to determine the positions of the cores. We utilized the equilibrated conformation of the star polymers from prior simulations and allowed the system to minimize its energy using the conjugate gradient algorithm to prevent any overlap between the monomers; during minimization, the cores remained fixed, and only arm monomers were free to move.

Subsequently, we permitted the system to equilibrate for a minimum of $10^6\sqrt{\frac{m\sigma^2}{\epsilon}}$, carefully ensuring that the simulation time exceeded the diffusion time for a single star polymer in a dilute concentration. In total, we conducted five replicas of each simulation.

In the Coarse-Grained Simulation, each star polymer is considered as a point-particle interacting through the following relation:

$$\frac{V(f,\lambda,r,\alpha)}{k_BT} = \begin{cases} \frac{2+9f^2}{24}(-\ln(r/\sigma)+\alpha) - f^2\lambda A(\lambda)e^{-\kappa(\lambda)r/\sigma}, & \text{if } r < 1, \\ \frac{2+9f^2}{24}\alpha e^{(1-r^2/\sigma^2)/2\alpha} - f^2\lambda A(\lambda)e^{-\kappa(\lambda)r/\sigma}, & \text{otherwise,} \end{cases} \tag{6}$$

where $A(\lambda) = 2.27-1.49\lambda$, and $\kappa(\lambda) = 6.56-7.61\lambda$, r is the distance between point particles, and σ is the length scale. This relation is obtained by fitting the simulation data from effective interaction. We carried out Monte Carlo (MC) simulations in the canonical ensemble for a system consisting of 1024 particles at different densities ($\varrho = 1024\sigma^2/L_xL_y$), with the particles interacting within a periodic 2D framework, the box size having $L_x : L_y = 2 : \sqrt{3}$ relation. The initial configuration was generated randomly. Before measuring the radial distribution function, the system was equilibrated for 10^6 MC cycles. For each functionality (f), solvent quality parameter (λ), density (ϱ), and tail parameter (α) five replicas were utilized.

In the next step, for looking at the geometrical aspect of star polymers, we conducted a comprehensive investigation into the shape deformation and interpenetration of star polymer

solutions. To facilitate our analysis, we utilized a sophisticated surface mesh technique that encloses each star polymer, enabling a detailed examination of these polymers in a continuous format. This methodology was employed through the application of the Alpha-shape method, which is implemented in the OVITO Python package.[3,4]

This approach began with the construction of a Delaunay tessellation derived from an extensive set of monomer coordinates, effectively decomposing the spatial domain into tetrahedral elements. For each tetrahedron formed within this tessellation, we computed the radius of its circumscribed sphere. Following this, any tetrahedron whose circumsphere radius exceeded a predetermined probe threshold of $r_p = 8\sigma$ was classified as a void, and the triangular facets associated with these void tetrahedra were systematically discarded. In this manner, we retained only those facets that served to separate void tetrahedra from solid tetrahedra (with a circumsphere radius $\leq r_p$). This circumsphere radius ensured the automatic sealing of all internal cavities smaller than the probe radius, ultimately resulting in a fully closed and watertight surface mesh characterized by an Euler characteristic of $\chi = 2$.

Subsequent to the generation of this surface mesh, we projected it onto the $x - y$ plane, thereby creating a polygon that encapsulates the relevant geometric features of the star polymer solutions. To analyze the geometric properties of this polygon, we employed the SHAPELY Python package,[5] which allowed us to accurately calculate both the overlap and the area of the resultant polygon.

Star Polymer Shape

In the main paper, we have used the Daude-Cotton blob model and numerical data to illustrate the coil-to-globule transformation of star polymers. As observed, as solvent conditions worsen, the polymer becomes increasingly compact. In Θ-solvent, we see a constant monomer density profile akin to what one observes in a melt. Further worsening of the solvent conditions leads to the buckling of star polymers and clustering of the arms on one side due to

these attractive forces. Consequently, star polymers exhibit a loss of angular symmetry in poor solvent conditions, adopting a highly anisotropic conformation, as illustrated in Figure S2. For $\lambda < \lambda_\Theta$, the polymers take on a disk shape; whereas for $\lambda > \lambda_\Theta$, a crescent or moon shape of the crystallized arm monomer is observed. Notably, this behavior was not observed in non-confined star polymers.[1]

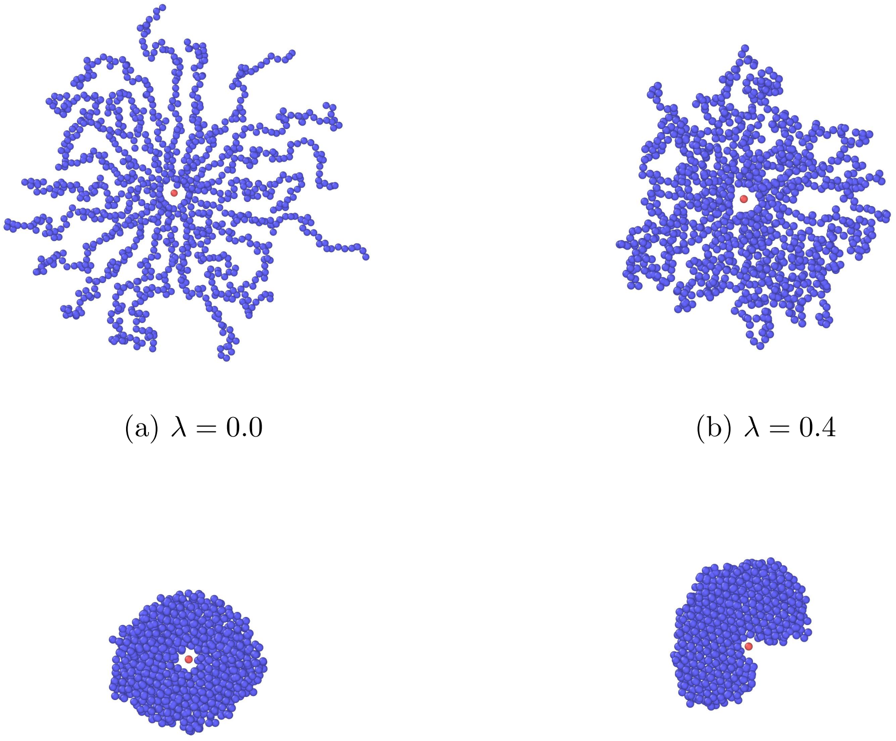

(a) $\lambda = 0.0$ (b) $\lambda = 0.4$

(c) $\lambda = 0.78$ (d) $\lambda = 1.0$

Figure S2: The figure depicts the coil-globule transition of star polymers. Upon reaching the Θ temperature, the star shifts from its disk shape to an anisotropic moon shape. Specifically, (a) shows a snapshot of the athermal star polymer; (b) presents the star polymer in a good solvent with $\lambda = 0.4$; (c) illustrates the polymer near the Θ temperature; and (d) captures the polymer in a bad solvent with $\lambda = 1.0$, demonstrating its anisotropic moon shape as it loses its disk-like form.

The observed shape anisotropy can be quantified using the gyration ellipsoid. Let $\mathbf{r}$ denote the positions of the star monomers in the center of the mass reference frame. We can

define the Gyration Tensor as follows:

$$\hat{G} = \frac{1}{Nf+1} \sum_{i=0}^{Nf} \mathbf{r}_i \otimes \mathbf{r}_i \tag{7}$$

The trace of this tensor corresponds to the radius of gyration. This tensor is of size 3×3 and has three principal moments denoted as $\lambda_1 \geq \lambda_2 \geq \lambda_3$. Utilizing these three eigenvalues, we can define shape parameters, particularly the relative anisotropy, which is expressed as follows:

$$\delta = 2\frac{\lambda_1^4 + \lambda_2^4}{\lambda_1^2 + \lambda_2^2} - 1 \tag{8}$$

In this context, $\delta \in [0, 1]$, where a value of 0 represents a perfectly circular shape. As δ increases, the degree of shape anisotropy also increases.

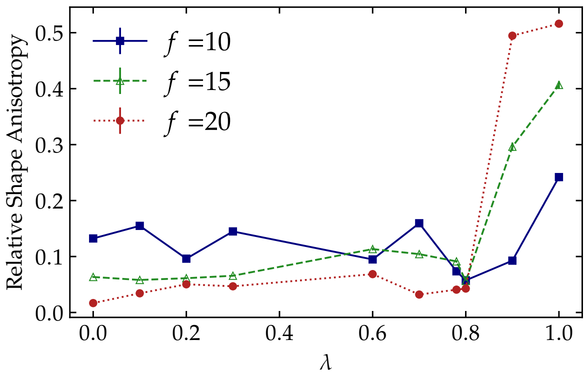

Figure S3: Anisotropy of the star polymers as a function of λ. The anisotropy increases with λ, indicating more anisotropic conformations in poorer solvents.

Figure S3 illustrates the anisotropy of star polymers as a function of solvent quality (λ). As λ increases from 0 to the λ_θ, we observe a consistent isotropic, disk-like shape, with circular symmetry becoming more pronounced as the number of arms increases, as anticipated. However, beyond the Θ temperature, there is a marked increase in polymer anisotropy, indicating a transition to a moon-like shape.

Athermal Stars, Effective Interaction

As indicated in the main text, the precise analytical form of the tails cannot be deduced by directly measuring the tails. For example, the following plot presents for $f = 10, 15$, we cannot exactly deduce the form of the tails.

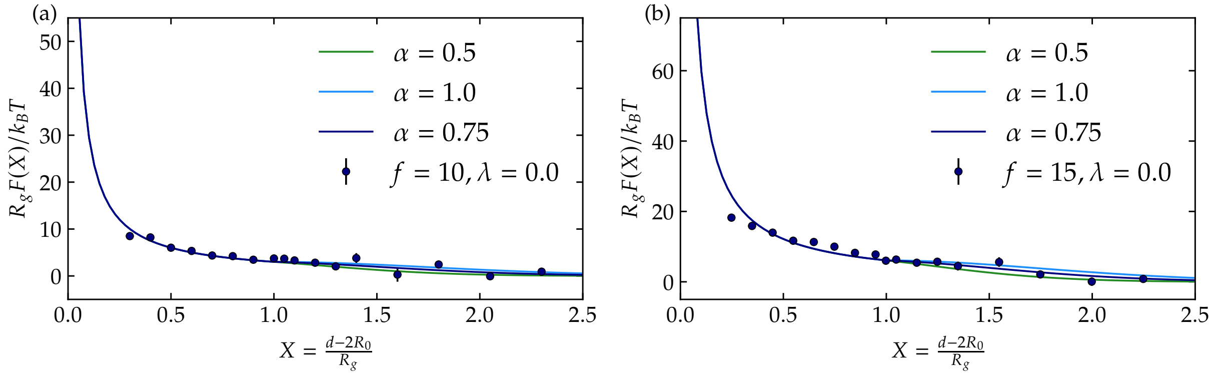

Figure S4: The relationship between normalized force and normalized core-to-core distance in athermal star polymers is presented herein. R_g represents the gyration radius of the polymers, and R_0 is the core size of the stars, d is the core-to-core distance between two-star polymers. The data obtained from molecular dynamics (MD) simulations are shown as closed circles, and the curves illustrate the resulting fits based on Equation (6) for various values of α.

Thermal Polymers, Effective Interaction

As outlined in the main text, we have meticulously calculated the exact shape of the interaction tail for each functionality and solvent quality. This was achieved through extensive simulations of 32 star polymers at a monomer-resolved level, as well as coarse-grained Monte Carlo simulations of point-like systems interacting according to the potential expressed in equation 6. By varying the α parameter of the tail, we identified the optimal α parameter as the one that exhibits the least deviation from the full monomer-resolved radial distribution function.

We have presented the radial distribution of athermal star polymers, analyzed at both the

monomer-resolved and coarse-grained levels. Our observations reveal that varying functionality leads to different values of α, and that even minor changes in the tail of the potential can yield misleading outcomes. In this work, we extend our findings to thermal polymers, demonstrating that the tail parameter is influenced by both solvent quality and functionality.

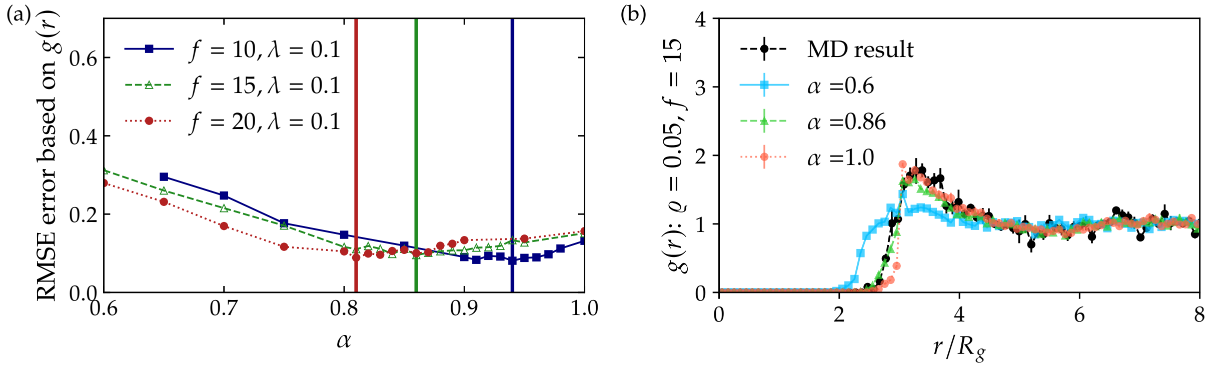

Figure S5: (a) presented the RMSE error calculated for thermal star polymers for $\lambda = 0.1$. (b) the $g(r)$ of monomers resolved simulation of themal star polymers (in black), and some coarse-grained simulation with effective interaction and different values of α, the green curve is presenting the selected α based on the RMSE error. As expected, The inset shows $\alpha \propto 1/\sqrt{f}$.

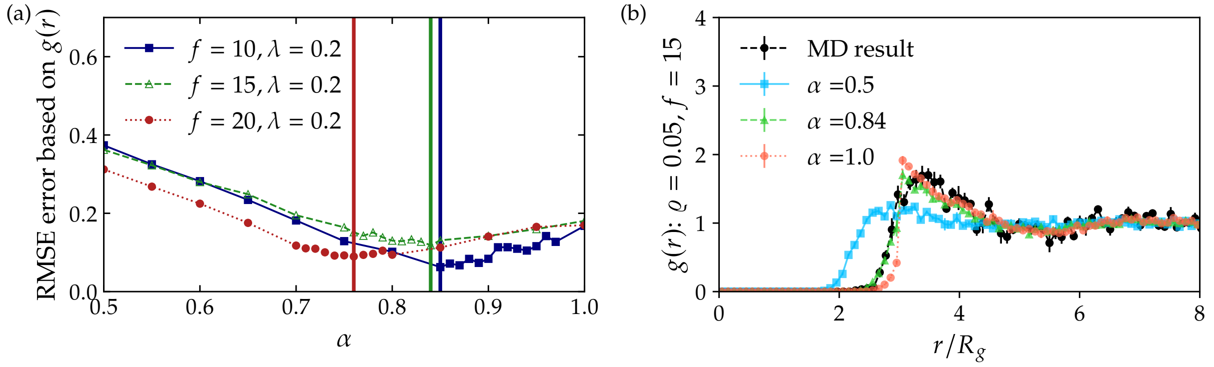

Figure S6: (a) presented the RMSE error calculated for thermal star polymers for $\lambda = 0.2$. (b) the $g(r)$ of monomers resolved simulation of themal star polymers (in black), and some coarse-grained simulation with effective interaction and different values of α, the green curve is presenting the selected α based on the RMSE error. As expected, The inset shows $\alpha \propto 1/\sqrt{f}$.

The result is presented in figures S5, to S8. The results are presented in Figures S5 to S8. It is clear that a decrease in solvent quality results in a reduction of the parameter α.

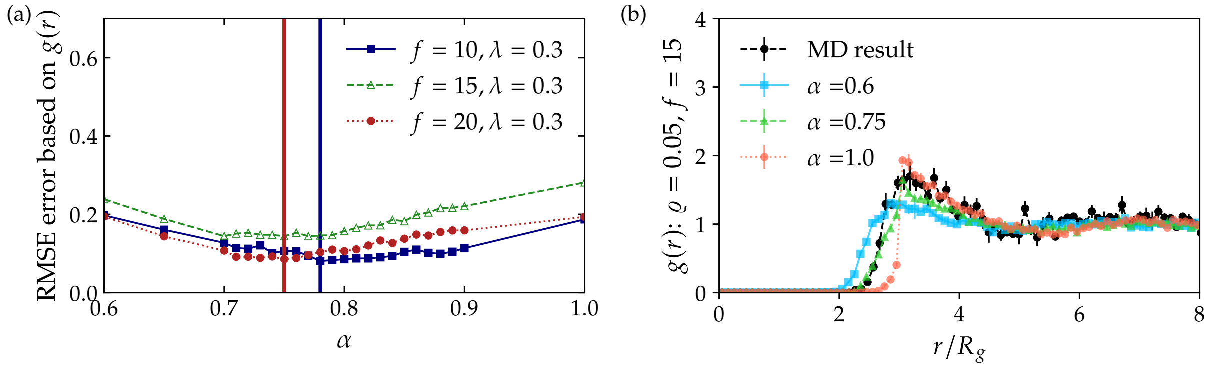

Figure S7: (a) presented the RMSE error calculated for thermal star polymers for $\lambda = 0.3$. (b) the $g(r)$ of monomers resolved simulation of themal star polymers (in black), and some coarse-grained simulation with effective interaction and different values of α, the green curve is presenting the selected α based on the RMSE error. As expected, The inset shows $\alpha \propto 1/\sqrt{f}$.

Notably, while the values of $A(\lambda)$ and $\kappa(\lambda)$ for $\lambda = 0.1, 0.2, 0.3$ were not calculated directly but rather derived through linear regression, they display a radial distribution function that closely matches that of the monomer-resolved simulations. Additionally, for $\lambda = 0.4$, we have incorporated the radial distribution at higher concentrations. The consistency between the results from the coarse-grained model and the monomer-resolved simulations highlights the success of the effective interaction in dense thermal solutions.

Concentrated solutions

Isotopic shrinkage of polymers in two dimensions

An important consideration in high polymer concentration is that polymers tend to shrink. This isotropic shrinking occurs independently of effective interactions. According to polymer theory, when the concentration exceeds the overlap concentration, a scaling law governs this isotropic shrinkage in three dimensions; it is well-established that this scaling follows the relationship $R \sim \varrho^{-(\nu-1/2)/(3\nu-1)}$.[6] We will apply a similar approach to determine the relationship in two dimensions.

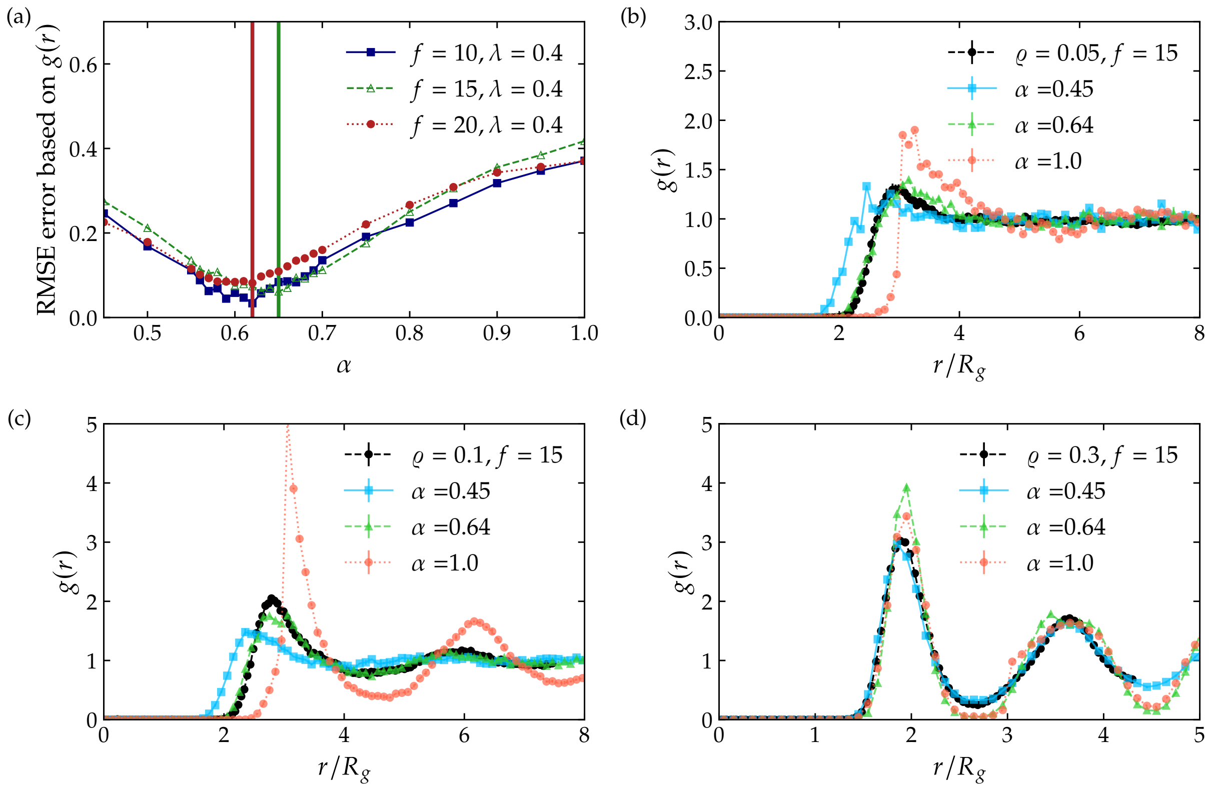

Figure S8: (a) presented the RMSE error calculated for thermal star polymers for $\lambda = 0.4$. (b) the $g(r)$ of monomers resolved simulation of themal star polymers (in black), and some coarse-grained simulation with effective interaction and different values of α, the green curve is presenting the selected α based on the RMSE error. The inset shows that the α has reached a value of 0.6 and cannot decreased further. (c) the same for density $\varrho = 0.1$, and (d) $\varrho = 0.3$, showing the calculated effective interaction and selected α, are working for densities as high as $\varrho = 0.3$.

To begin, we will examine the Flory excluded volume interaction energy. We define v as the excluded volume, presuming that we are focusing on good solvents, where $v > 0$. The size of the thermal blob is denoted as ξ_T, with each blob containing g_T monomers in a random walk conformation. Thus, we have the following relationships:

$$\begin{cases} \xi_T \sim b\sqrt{g_T} \\ k_B T v(\frac{g_T}{\xi_T})^2 \sim k_B T \end{cases} \Rightarrow \begin{cases} g_T \sim b^2/v. \\ \xi_T \sim b^2/\sqrt{v}. \end{cases} \tag{9}$$

Here, b represents the Kuhn length. For scales larger than the thermal blob, we find:

$$R \sim \left(\frac{N}{g_T}\right)^2 \xi_T \rightarrow R \sim N^\nu b^{2-2\nu}\sqrt{v^{2\nu-1}}. \tag{10}$$

At high concentrations, the correlation length (ξ_c) is defined as the length scale at which the high concentration of the solution does not influence a polymer. As a result, we can consider the system to be composed of space-filling correlation blobs. Each blob contains g_c monomers and exhibits a conformation similar to that of a polymer in a dilute solution, which is described by Equation 10:

$$\xi_c \sim g_c^\nu b^{2-2\nu}\sqrt{v^{2\nu-1}}. \tag{11}$$

At large scale the system is a melt of these correlation blobs. Thus, we have $R \sim \xi_c\sqrt{N/g_c}$. Given that the system maintains a uniform concentration, we can treat the concentration of monomers within a blob as the effective concentration, leading to the relation $\varrho = \frac{g_c b^2}{\xi_c^2}$. This reasoning yields the following result:

$$R \sim \varrho^{-1/2}. \tag{12}$$

Unlike in the three-dimensional case, this intriguing result is independent of both ν and v.

Polymers in two dimensions tend to shrink more strongly than those in three dimen-

sions, which is expected, as reducing dimensionality increases the effect of excluded volume interactions.

Overlapping Star polymers

Herein, we present overlapping parameter star polymers for two different surface meshes constructed: one that encapsulates all monomers and one that considers only monomers in the core region. The results are presented successfully in figures S9, and S10.

A key observation is that when we exclude the soft shell from consideration, all functionalities behave similarly in terms of overlapping. Thus, the increased overlapping of the soft polymers arises solely from the contribution of the soft shell. Additionally, we note a significant level of overlapping that our analytical model cannot account for. By examining the snapshot, it becomes apparent that this excess overlapping originates from the highly sparse regions in the outer areas of the meshes constructed with all monomers.

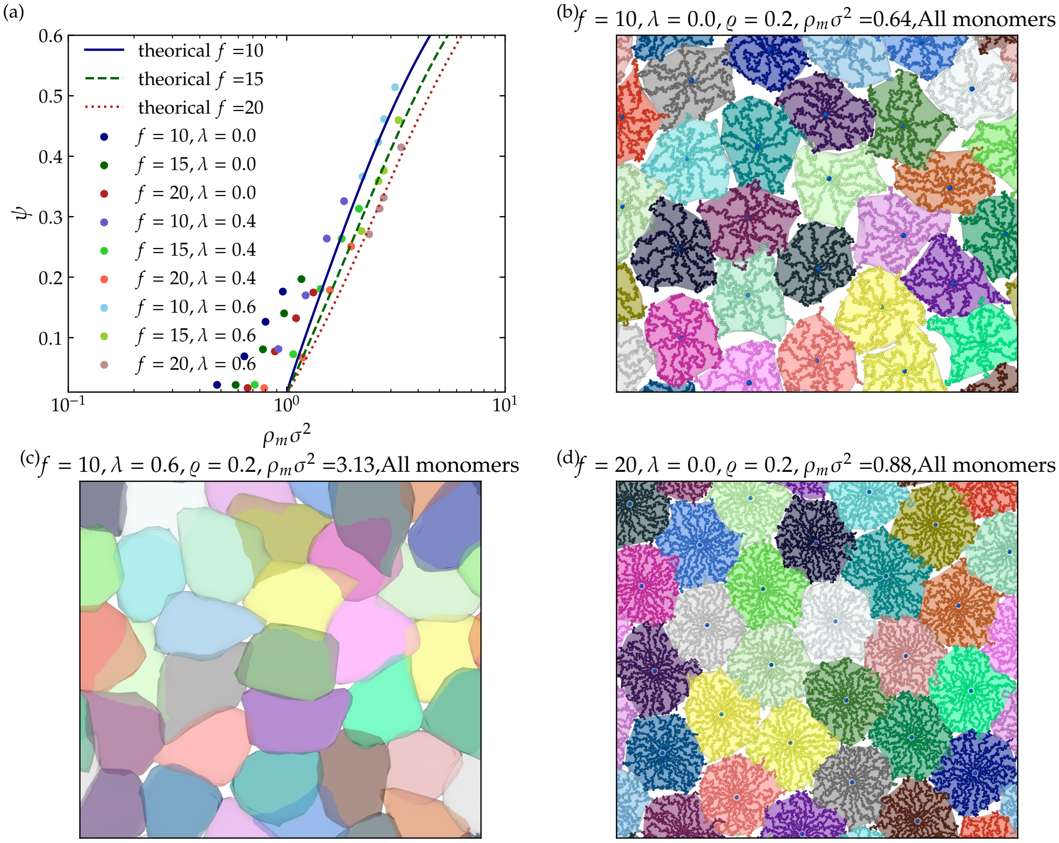

Figure S9: (a) Overlap parameter ψ computed analytically (lines) and measured from simulations (symbols) as a function of the monomer density, for meshes constructed from all monomers. (b)–(d) Projected snapshots of the polymer surface mesh illustrating overlap for representative state points at fixed star concentration $\varrho = 0.2$: (b) $f = 10$, $\lambda = 0.0$, ($\rho_m\sigma^2 = 0.64$); (c) $f = 10$, $\lambda = 0.6$, ($\rho_m\sigma^2 = 3.13$); (d) $f = 20$, $\lambda = 0.0$, ($\rho_m\sigma^2 = 0.88$).

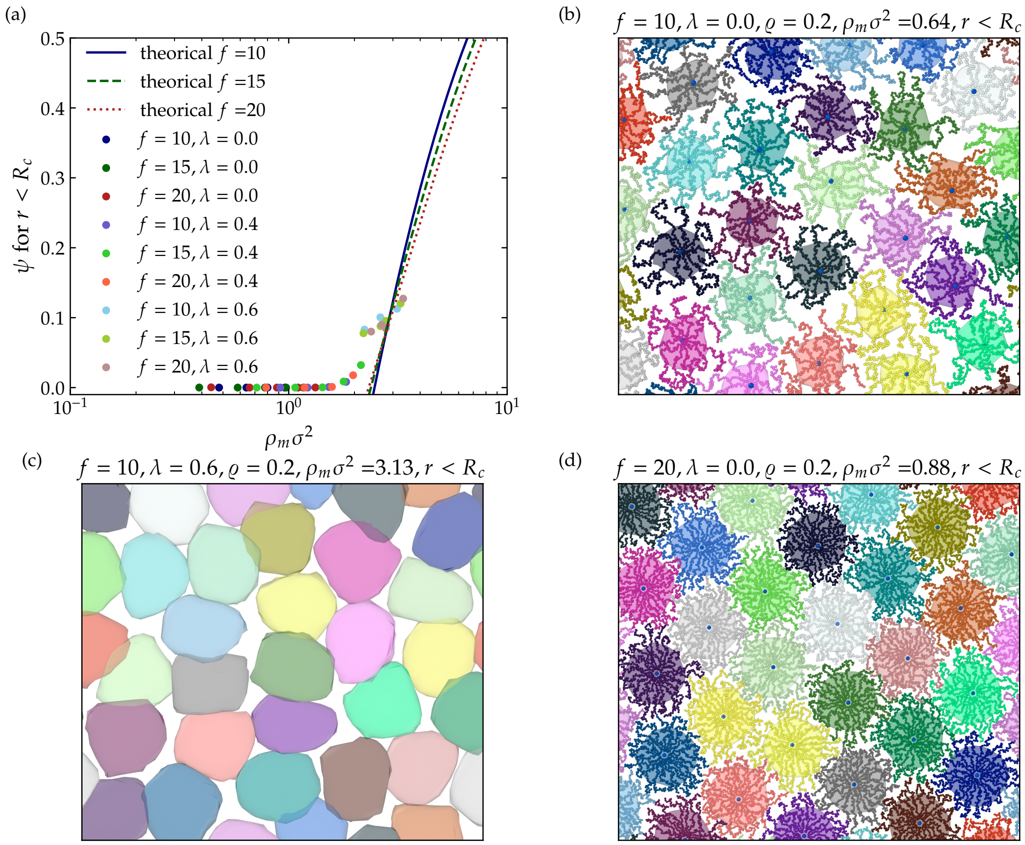

Figure S10: (a) Overlap parameter ψ computed analytically (lines) and measured from simulations (symbols) as a function of the monomer density, for meshes constructed from core section os star polymers. (b)–(d) Projected snapshots of the polymer surface mesh illustrating overlap for representative state points at fixed star concentration $\varrho = 0.2$: (b) $f = 10$, $\lambda = 0.0$, ($\rho_m\sigma^2 = 0.64$); (c) $f = 10$, $\lambda = 0.6$, ($\rho_m\sigma^2 = 3.13$); (d) $f = 20$, $\lambda = 0.0$, ($\rho_m\sigma^2 = 0.88$).

References

(1) Huissmann, S.; Blaak, R.; Likos, C. N. Star polymers in solvents of varying quality. *Macromolecules* **2009**, *42*, 2806–2816, DOI: 10.1021/ma8023359.

(2) Thompson, A. P.; Aktulga, H. M.; Berger, R.; Bolintineanu, D. S.; Brown, W. M.; Crozier, P. S.; in 't Veld, P. J.; Kohlmeyer, A.; Moore, S. G.; Nguyen, T. D.; Shan, R.; Stevens, M. J.; Tranchida, J.; Trott, C.; Plimpton, S. J. LAMMPS - a flexible simulation tool for particle-based materials modeling at the atomic, meso, and continuum scales. *Comput. Phys. Commun.* **2022**, *271*, 108171, DOI: 10.1016/j.cpc.2021.108171.

(3) Stukowski, A. Visualization and analysis of atomistic simulation data with OVITO–the Open Visualization Tool. *Model. Simul. Mater.* **2009**, *18*, 015012.

(4) Stukowski, A. Computational analysis methods in atomistic modeling of crystals. *J. Met.* **2014**, *66*, 399–407.

(5) Gillies, S.; others Shapely: manipulation and analysis of geometric objects. 2007–; https://github.com/Toblerity/Shapely.

(6) Rubinstein, M.; Colby, R. H. *Polymer physics*; Oxford university press, 2003.
